# Supplementary material for: Food purchase patterns in Nairobi before, during, and after the COVID-19 pandemic lockdown measures
Source: PLOS Glob Public Health. 2026 Jun 1;6(6):e0006544. doi: 10.1371/journal.pgph.0006544 (PMC13225382; doi:10.1371/journal.pgph.0006544)
Supplement: S1 Appendix — (DOCX) [file pgph.0006544.s001.docx]

**S1 Appendix: Classified food items in the data according to NOVA**

| **NOVA food classification** | **Food Items** |
| --- | --- |
| Processed Culinary Ingredients | Brown Sugar, Coconut Oil, Corn Oil, Ghee, Glucose, Herbal Salt, Honey, Icing Sugar, Jaggery, Maple Syrup, Mustard Seed Oil, Olive Oil, Rock Salt, Salted Butter, Sea Salt, Sesame Oil, Sunflower Oil, Table Salt, Unsalted Butter, Vegetable Fat, Vegetable Oil, White Sugar |
| Processed foods | Achari, Camembert Cheese, Canned Appricot, Canned Bamboo Shoot, Canned Beans, Canned Cherries, Canned Chicken, Canned Chickpeas, Canned Chilli, Canned Cucumber, Canned Fruit Cocktail, Canned Githeri, Canned Lychees, Canned Mixed Vegetables, Canned Mushrooms, Canned Olives, Canned Oranges, Canned Peach, Canned Pear, Canned Peas, Canned Pineapple, Canned Sardines, Canned Sweet Corn, Canned Sweet Kernel, Canned Tomatoes, Canned Tuna, Chedder Cheese, Cheese Spread, Dill Pickle, Feta Cheese, Gherkins Pickle, Gouda Cheese, Luncheon Meat, Mango Pickle, Mix Pickle, Mozzarella Cheese, Non-Alcoholic Malt Drinks, Paneer Cheese, Parmesan Cheese, Processed Cheese, Processed Cheese Slices, Salted Cashew Nuts, Salted Hazelnuts, Salted Macadamia Nuts, Salted Mixed Nuts, Salted Peanuts, Salted Peeled Peanuts, Salted Pistachio Nuts, Salted Skinned Peanuts, Sugared Cashew Nuts, Sugared Macadamia Nuts, Sugared Mixed Nuts, Sugared Peanuts, Tomato Paste |
| Ultra-processed foods | Almond Butter, Almond Milk, Almond Milk - Lactose Free, Artificial Sweeteners, Baby Foods - Puree, Baby/Infant Wheat Cereals, Baked Multigrain Wavy Chips, Baked Potato Bites, Baked Potato Chipsticks, Baked Potato Crackers, Baking Powder, Baking Soda, Baobab Seed Candy/Mabuyu, Barbecue Sauce, Beef Brawn, Beef Sausages/Smokies/Vienna, Biscuit Spread, Biscuits - Chocolate, Biscuits - Chocolate With Oats, Biscuits - Cream, Biscuits - Cream Crackers, Biscuits - Digestive, Biscuits - Digestive With Oats, Biscuits - Gingernut, Biscuits - Rusks, Biscuits - Shortbread, Biscuits - Sweet, Bottled Ice Coffee, Bottled Ice Teas, Bran Flake Cereals, Bread Crumbs, Brioche Bread, Brown Bread, Brown Sauce, Cake Mixes, Cakes, Candy Bar - Chocolate, Candy Bar - Non Chocolate, Caramel Sauce, Caramel Syrup, Cashewnut Milk, Cereal Bars, Cereal Straws, Chamoy Sauce, Cheese Cake, Chevda/Bombay Mix, Chewing Gums, Chicken Brawn, Chicken Burger, Chicken Pate, Chicken Sausages/Viennas, Chilli/Hot Sauce, Chocolate, Chocolate Bar, Chocolate Sauce, Chocolate Spreads, Chocolate Syrup, Choma Sauce, Cocoa - Sweetened With Milk, Cocoa - Sweetened Without Milk, Cocoa - Unsweetened Without Milk, Coconut Almond Milk, Coconut Burfi/Kashata, Coconut Cream, Coconut Cream Powder, Coconut Milk, Cookies, Cooking Cream, Cordials, Cordials Powder, Corn Balls, Corn Chips, Corn Curls, Corn Flake Cereals, Corn Puffs, Corn Rings, Cream Caramel, Cream Pudding, Cupcakes, Curacoa Syrup, Curry Paste, Custard, Custard Powder, Diet Shakes, Energy Drinks, Fish Sauce, Flavoured Fermented Milk, Flavoured Milk, Flavoured/Fruit Yoghurt, Food Colour Additives, Food Flavour Additives, French Bread, Fried Lentils, Fried Peas, Fruit Cereal Bars, Fruit Drinks/Sweetened Juice, Fruit Flavoured Cereals, Fruit Jam, Fruit Syrup, Ganthia Snacks, Gelatin Powder, Gelatin/Jelly Powder, Glucose Syrup, Golden Syrup, Granola Cereals, Gummy Candy, Halva/Halwa, Hazelnut Butter, Hazelnut Milk, Hazelnut Syrup, Horseradish Sauce, Ice Cream - Caramel, Ice Cream - Chocolate, Ice Cream - Other Flavours, Ice Cream - Strawberry, Ice Cream - Vanilla, Ice Cream - Vanilla/Strawberry, Ice Cream Powder, Infant Formula, Instant Cereal, Instant Soup - Chicken, Instant Soup - Chicken & Mushroom, Instant Soup - Minestrone, Instant Soup - Mushroom, Instant Soup - Onion, Instant Soup - Tomato, Instant Soup - Vegetable, Isotonic Drinks, Jhal Muri, Labania/Peanut Brittle, Lollipops, Mango Chutney, Marble Cakes, Margarine, Marmalade, Marshmallow, Mayonnaise, Mint Sauce, Mixed Fruit Chutney, Muffins, Mustard Sauce, Non-Dairy Ice Fruit, Noodles, Oat & Wheat Cereal Biscuits, Oat & Wheat Cereals, Oat Almond Milk, Oat Milk, Oat Milk - Lactose Free, Olive Chutney, Onion Rings, Organic Drinks, Oyster Sauce, Pasta Sauce, Pastries - Chocolate Balls, Pastries - Croissant, Pastries - Custard Tart, Pastries - Doughnut, Pastries - Galette, Pastries - Puff, Pastries - Scones, Peanut Butter, Pizza Sauce, Plain Buns, Plain Rolls, Popcorn, Potato Crisps, Potato Sticks, Pre-Prepared Chicken Sausage, Pre-Prepared Pie, Pre-Prepared Vegetable Burger, Pretzels, Pudding Powder, Puffed Corn Cereals, Puffed Wheat Cereals, Queencakes, Ready To Heat Beef Burger, Ready To Heat Beef Samosa, Ready To Heat Beef Spring Rolls, Ready To Heat Cheese Samosa, Ready To Heat Chicken Samosa, Ready To Heat Flaky Parathas, Ready To Heat Lasagna, Ready To Heat Mutton Samosa, Ready To Heat Shrimps, Ready To Heat Spring Rolls, Ready To Heat Vegetable Samosa, Ready To Heat Veggie Fingers, Ready To Vegetable Spring Rolls, Rice Cake, Rice Flake Cereals, Rose Syrup, Salad Cream, Sandwich Spread, Sesame Buns, Sesame/Simsim Bar, Smarties, Sodas, Soup Flavour Beef Cubes, Soup Flavour Beef Mixes, Soup Flavour Chicken Cubes, Soup Flavour Chicken Mixes, Soup Flavour Cubes, Soup Flavour Mixes, Soup Flavour Tomato & Onion Cubes, Soup Flavour Tomato Ginger & Garlic Mixes, Soup Flavour Vegetable Cubes, Sour Cream Dips, Soy Sauce, Soya Milk, Soya Milk - Lactose Free, Sparkling Non-Alcoholic Wine, Squashes, Sweet Buns, Sweet Rolls, Sweets/Candy, Sweets/Candy - Chocolate, Table Syrup, Tahina, Tamarid Sauce, Tarmarind Chutney, Tartare Sauce, Tomato Ketchup, Tomato Sauce, Topping Sauces, Tortilla Wraps, Vanilla Syrup, Vegan Creams, Vermicelli/Sprinkles, Vinegar, Wafers, Wafers - Chocolate, Wheat Biscuit Cereals, Wheat Cereals, Wheat Flake Cereals, Whipped Cream Powder, White Bread, Whole Grain Cereals, Whole Grain Oats Cereals, Whole Grain Wheat Cereals, Whole Sweetened Condensed Milk, Worcestershire Sauce, Yeast |
| Unprocessed/Minimally processed foods | Alkaline Water, Almond Flakes, Almond Flour, Apples, Baby/Infant Porridge Flour, Beef Mince, Black Beans (Njahi), Blue Berries, Brown Rice, Cassava Flour, Chamomile Tea, Chick Peas, Chick Peas Split, Chicken, Chicken Giblets, Coconut Water, Coffee - Sweetened With Milk, Coffee - Sweetened Without Milk, Coffee - Unsweetened With Milk, Coffee - Unsweetened Without Milk, Corn Flour, Cous Cous, Desiccated Coconut, Dried Apricots, Dried Dates, Dried Figs, Dried Grated Coconut, Dried Mixed Fruits And Nuts, Dry Peas, Eggs, Fenugreek Seeds, Fish - Tilapia, Flavoured Water, Flax/ Linseeds, Fresh Cream, Fresh/Fruit Juices - No Added Sugar, Frozen Broccoli, Frozen Raw Potato Chips, Gram Flour, Granola, Grapes, Green Grams, Green Grams Split, Green Peas, Green Tea, Green Tea Drink, Herbal Tea, Herbs - Basil, Herbs - Bay Leaves, Herbs - Black Maca, Herbs - Chia Seeds, Herbs - Coriander/Dhania, Herbs - Dill Seeds, Herbs - Fennel Powder, Herbs - Fennel Seeds, Herbs - Marjoram, Herbs - Mint, Herbs - Mixed Herbs, Herbs - Moringa, Herbs - Oregano, Herbs - Parsley, Herbs - Psylluim, Herbs - Rosemary, Herbs - Sage, Herbs - Stevia, Herbs - Thyme, Hibiscus Tea, Kidney Beans, Kiwi Fruit, Lactose Free Milk, Lemon Tea, Lentils, Lima (Butter) Beans, Long Life Skimmed Milk, Long Life/Uht Whole Milk, Maize Flour, Mango, Mineral Water, Mix Vegetables, Mixed Vegetables, Muesli, Muesli Cereals, Natural/Plain Fermented Milk, Natural/Plain Yoghurt, Oat Bran, Oats, Oranges, Pasta - Grandi, Pasta - Lasagne, Pasta - Macaroni, Pasta - Spaghetti, Peanut Powder, Peppermint Tea, Popcorn Kernels, Porridge Flour - Finger Millet, Sorghum & Maize, Porridge Flour - Maize & Soya, Porridge Flour - Millet, Porridge Flour - Sorghum, Porridge Flour - Wheat, Prunes/Dried Plums, Pumpkin Seeds, Quinoa, Quinoa Seeds, Raisins, Raspberry, Raw Almonds, Raw Brazil Nuts, Raw Cashew Nuts, Raw Hazelnuts, Raw Peanuts, Raw Pecan Nuts, Raw Pine Nuts, Raw Pistachio Nuts, Raw Walnuts, Red Millet Flour, Red Rice, Rice Flour, Ripe Bananas, Semi-Skimmed Fresh Milk, Sesame Seeds, Skimmed Fresh Milk, Skimmed Milk Powder, Soya Flour, Spices - Beef Masala, Spices - Black Pepper, Spices - Cardamon, Spices - Cayenne Pepper, Spices - Chicken Masala, Spices - Chillies, Spices - Chips Masala Seasoning, Spices - Cinnamon, Spices - Citric Acid, Spices - Cloves, Spices - Cumin, Spices - Curry Powder, Spices - Dhana Jeera, Spices - Fish Masala, Spices - Food Seasoning, Spices - Garam Masala, Spices - Garlic, Spices - Ginger, Spices - Ginger & Garlic, Spices - Githeri Masala, Spices - Meat Seasoning, Spices - Meat Tenderizer, Spices - Mixed Spices, Spices - Mustard Powder, Spices - Nutmeg, Spices - Onion Powder, Spices - Paprika, Spices - Peri Peri Seasoning, Spices - Pilau Masala, Spices - Pilau Mix, Spices - Pizza Seasoning, Spices - Salad Seasoning, Spices - Tandoori Masala, Spices - Tea Masala, Spices - Turmeric, Spices - White Pepper, Spring Water, Strawberries, Sultanas, Sunflower Seeds, Sweet Potato Flour, Tea - Sweetened With Milk, Tea - Unsweetened With Milk, Tea - Unsweetened Without Milk, Terere Flour, Terere/Mchicha/Amaranthus, Wheat, Wheat - Bulgur, Wheat -Whole, Wheat Flour, Wheat Flour - Atta, Wheat Grass, Whipped Cream, White Rice, Whole Evaporated Unsweetened Milk, Whole Fresh Camel Milk, Whole Fresh Milk, Whole Milk Powder |
