# Supplementary material for: Food purchase patterns in Nairobi before, during, and after the COVID-19 pandemic lockdown measures
Source: PLOS Glob Public Health. 2026 Jun 1;6(6):e0006544. doi: 10.1371/journal.pgph.0006544 (PMC13225382; doi:10.1371/journal.pgph.0006544)
Supplement: S1 Table — (DOCX) [file pgph.0006544.s002.docx]

**S1 Table: Parameter estimates, confidence intervals, and Z-test p-values from the full ITS-ARIMA models predicting the weekly proportion of NOVA classification**

| **Variable** | **Category** | **Optimal ITS- ARIMA model** | **Ljung-Box Pierce Test p-value** | **Intercept (β_0_)** | | **Pre-COVID (β_1_)** | | **Start of Lockdown (β_2_)** | | **COVID Period (β_3_)** | | **End of Lockdown (β_4_)** | | **Post-COVID (β_5_)** | |
| --- | --- | --- | --- | --- | --- | --- | --- | --- | --- | --- | --- | --- | --- | --- | --- |
|  |  |  |  | **Coefficient**  **(95% CI)** | **Z test  p-value** | **Coefficient**  **(95% CI)** | **Z test  p-value** | **Coefficient**  **(95% CI)** | **Z test  p-value** | **Coefficient**  **(95% CI)** | **Z test  P-value** | **Coefficient**  **(95% CI)** | **Z test  p-value** | **Coefficient**  **(95% CI)** | **Z test  p-value** |
| NOVA food classification | Processed Culinary Ingredients | ARIMA(5,0,0) errors | 0.948 | 1.8871  (1.6853, 2.0890) | **<0.001** | -0.0047  (-0.0223, 0.0129) | 0.600 | 0.2271  (0.0148, 0.4394) | **0.036** | 0.0035  (-0.0150, 0.0220) | 0.711 | 0.1454  (-0.1202, 0.4110) | 0.283 | -0.0011  (-0.0147, 0.0125) | 0.876 |
|  | Processed foods | ARIMA(1,0,0) errors | 0.914 | 2.0114  (1.6572, 2.3656) | **<0.001** | 0.0029  (-0.0096, 0.0154) | 0.648 | 0.4153  (-0.0340, 0.8647) | 0.070 | 0.0032  (-0.0120, 0.0185) | 0.680 | 0.1921  (-0.2465, 0.6308) | 0.391 | -0.0140  (-0.0286, 0.0007) | 0.062 |
|  | Ultra-processed foods | ARIMA(0,0,3) errors | 0.615 | 74.2952  (72.9282, 75.6622) | **<0.001** | 0.0322  (0.0094, 0.0550) | **0.006** | -2.4716  (-4.2736, -0.6695) | **0.007** | -0.0241  (-0.0642, 0.0160) | 0.239 | -0.2190  (-2.0137, 1.5757) | 0.811 | 0.0103  (-0.0297, 0.0503) | 0.614 |
|  | Unprocessed/Minimally processed foods | ARIMA(0,0,3) errors | 0.681 | 21.7769  (20.5919, 22.9620) | **<0.001** | -0.0295  (-0.0500, -0.0090) | **0.005** | 1.6733  (0.1210, 3.2255) | **0.035** | 0.0180  (-0.0171, 0.0531) | 0.315 | -0.1601  (-1.6936, 1.3734) | 0.838 | 0.0030  (-0.0320, 0.0381) | 0.867 |
| Note: Mixed Dishes and Fast Foods/Starchy Roots and Tubers Transactions omitted in ITS analysis as data points limited in duration and coverage | | | | | | | | | | | | | | |  |
