# Supplementary material for: Food purchase patterns in Nairobi before, during, and after the COVID-19 pandemic lockdown measures
Source: PLOS Glob Public Health. 2026 Jun 1;6(6):e0006544. doi: 10.1371/journal.pgph.0006544 (PMC13225382; doi:10.1371/journal.pgph.0006544)
Supplement: S2 Table — (DOCX) [file pgph.0006544.s003.docx]

**S2 Table: Parameter estimates, confidence intervals, and Z-test p-values from the full ITS-ARIMA models predicting the weekly mean nutrient values per 100g/100ml of food**

| **Variable** | **Category** | **Optimal ITS- ARIMA model** | **Ljung-Box Pierce Test p-value** | **Intercept (β_0_)** | | **Pre-COVID (β_1_)** | | **Start of Lockdown (β_2_)** | | **COVID Period (β_3_)** | | **End of Lockdown (β_4_)** | | **Post-COVID (β_5_)** | |
| --- | --- | --- | --- | --- | --- | --- | --- | --- | --- | --- | --- | --- | --- | --- | --- |
|  |  |  |  | **Coefficient**  **(95% CI)** | **Z test  p-value** | **Coefficient**  **(95% CI)** | **Z test  p-value** | **Coefficient**  **(95% CI)** | **Z test  p-value** | **Coefficient**  **(95% CI)** | **Z test  p-value** | **Coefficient**  **(95% CI)** | **Z test  p-value** | **Coefficient**  **(95% CI)** | **Z test  p-value** |
| Proximates | Energy (kcal) | ARIMA(1,0,0) errors | 0.413 | 534.0219  (510.1383, 557.9056) | **<0.001** | 0.6265  (0.2796, 0.9734) | **<0.001** | 5.7608  (-25.4839, 37.0055) | 0.718 | -0.0511  (-0.7256, 0.6235) | 0.882 | -7.1228  (-38.1618, 23.9162) | 0.653 | -0.7579  (-1.4327, -0.0830) | **0.028** |
|  | Water (g) | ARIMA(5,0,0) errors | 0.966 | 39.0686  (37.4957, 40.6416) | **<0.001** | -0.0091  (-0.0312, 0.0130) | 0.418 | -0.7891  (-2.4119, 0.8336) | 0.341 | 0.0137  (-0.0298, 0.0572) | 0.536 | 0.1350  (-1.4775, 1.7475) | 0.870 | -0.0095  (-0.0531, 0.0340) | 0.668 |
|  | Protein (g) | ARIMA(1,0,0) errors | 0.642 | 6.2559  (6.1874, 6.3245) | **<0.001** | -0.0037  (-0.0047, -0.0027) | **<0.001** | 0.2738  (0.1746, 0.3729) | **<0.001** | 0.0027  (0.0007, 0.0046) | **0.008** | 0.0677  (-0.0307, 0.1661) | 0.177 | 0.0015  (-0.0005, 0.0035) | 0.133 |
|  | Fat (g) | ARIMA(1,0,2) errors | 0.911 | 11.4115  (11.0230, 11.8000) | **<0.001** | 0.0011  (-0.0045, 0.0067) | 0.704 | 0.2717  (-0.2074, 0.7507) | 0.266 | 0.0098  (-0.0010, 0.0206) | 0.074 | 0.0539  (-0.4092, 0.5169) | 0.820 | -0.0200  (-0.0309, -0.0091) | **<0.001** |
|  | Carbohydrate available (g) | ARIMA(3,0,2) errors | 0.637 | 43.2752  (42.1291, 44.4212) | **<0.001** | 0.0121  (-0.0042, 0.0284) | 0.147 | -0.4061  (-1.7169, 0.9047) | 0.544 | -0.0134  (-0.0453, 0.0184) | 0.407 | -0.1434  (-1.4542, 1.1673) | 0.830 | 0.0148  (-0.0169, 0.0466) | 0.360 |
|  | Fibre (g) | ARIMA(0,0,4) errors | 0.875 | 4.0969  (3.8900, 4.3038) | **<0.001** | -0.0030  (-0.0060, 0.0001) | 0.054 | 0.4676  (0.1754, 0.7599) | **0.002** | 0.0016  (-0.0043, 0.0075) | 0.592 | 0.0185  (-0.2651, 0.3021) | 0.898 | -0.0003  (-0.0062, 0.0056) | 0.921 |
|  | Cholesterol (mg) | ARIMA(1,0,1) errors | 0.927 | 20.4379  (18.9068, 21.9690) | **<0.001** | 0.0148  (-0.0068, 0.0363) | 0.180 | 1.0348  (-0.5803, 2.6498) | 0.209 | -0.0223  (-0.0648, 0.0203) | 0.305 | 1.1801  (-0.4984, 2.8587) | 0.168 | -0.0044  (-0.0468, 0.0381) | 0.841 |
| Minerals | Calcium (mg) | ARIMA(0,0,4) errors | 0.823 | 96.2366  (92.3680, 100.1053) | **<0.001** | -0.0096  (-0.0667, 0.0475) | 0.742 | 8.8285  (3.3571, 14.3000) | **0.002** | -0.0881  (-0.1974, 0.0211) | 0.114 | 0.8640  (-4.3165, 6.0445) | 0.744 | 0.0513  (-0.0593, 0.1618) | 0.363 |
|  | Iron (mg) | ARIMA(1,0,0) errors | 0.673 | 2.2175  (2.1383, 2.2966) | **<0.001** | -0.0020  (-0.0031, -0.0008) | **0.001** | 0.1635  (0.0478, 0.2792) | **0.006** | 0.0010  (-0.0012, 0.0033) | 0.371 | 0.0415  (-0.0735, 0.1564) | 0.479 | 0.0009  (-0.0014, 0.0032) | 0.430 |
|  | Magnesium (mg) | ARIMA(4,0,0) errors | 0.858 | 34.9134  (33.7241, 36.1028) | **<0.001** | -0.0213  (-0.0386, -0.0040) | **0.016** | 3.1270  (1.5434, 4.7106) | **<0.001** | 0.0092  (-0.0246, 0.0430) | 0.593 | 0.4964  (-1.0575, 2.0503) | 0.531 | 0.0068  (-0.0271, 0.0407) | 0.694 |
|  | Phosphorus (mg) | ARIMA(0,0,4) errors | 0.815 | 135.7153  (130.8427, 140.5879) | **<0.001** | -0.0004  (-0.0721, 0.0714) | 0.992 | 11.1576  (4.2947, 18.0206) | **0.001** | -0.0786  (-0.2173, 0.0601) | 0.267 | 2.8817  (-3.8175, 9.5808) | 0.399 | 0.0088  (-0.1307, 0.1482) | 0.902 |
|  | Potassium (mg) | ARIMA(0,0,5) errors | 0.960 | 306.9143  (298.5621, 315.2665) | **<0.001** | -0.1714  (-0.2950, -0.0478) | **0.007** | 28.3732  (16.3004, 40.4459) | **<0.001** | 0.0538  (-0.1852, 0.2928) | 0.659 | 0.2298  (-11.5398, 11.9994) | 0.969 | -0.0706  (-0.3107, 0.1696) | 0.565 |
|  | Sodium (mg) | ARIMA(3,0,2) errors | 0.441 | 338.1077  (316.5789, 359.6365) | **<0.001** | -0.5714  (-0.8871, -0.2556) | **<0.001** | 55.1169  (24.2455, 85.9882) | **<0.001** | -0.2410  (-0.8786, 0.3965) | 0.459 | 19.9590  (-13.0832, 53.0013) | 0.236 | 0.7997  (0.1737, 1.4257) | **0.012** |
|  | Zinc (mg) | ARIMA(0,0,4) errors | 0.863 | 0.7540  (0.7378, 0.7701) | **<0.001** | -0.0001  (-0.0004, 0.0002) | 0.472 | 0.0421  (0.0187, 0.0654) | **<0.001** | 0.0001  (-0.0004, 0.0005) | 0.791 | 0.0132  (-0.0094, 0.0357) | 0.253 | 0.0000  (-0.0005, 0.0004) | 0.894 |
|  | Selenium (mcg) | ARIMA(1,0,0) errors | 0.688 | 6.6216  (6.5160, 6.7271) | **<0.001** | -0.0044  (-0.0060, -0.0029) | **<0.001** | 0.1951  (0.0415, 0.3486) | **0.013** | 0.0059  (0.0029, 0.0090) | **<0.001** | 0.1900  (0.0365, 0.3435) | **0.015** | -0.0001  (-0.0032, 0.0029) | 0.931 |
| Vitamins | Vitamin A-RE (mcg) | ARIMA(4,0,0) errors | 0.990 | 163.7523  (152.4545, 175.0500) | **<0.001** | -0.4306  (-0.5944, -0.2668) | **<0.001** | 17.1901  (3.3823, 30.9978) | **0.015** | 0.2392  (-0.0654, 0.5439) | 0.124 | 4.0687  (-9.3636, 17.5011) | 0.553 | 0.1548  (-0.1409, 0.4505) | 0.305 |
|  | Thiamin (mg) | ARIMA(3,0,0) errors | 0.988 | 0.1827  (0.1801, 0.1853) | **<0.001** | -0.0002  (-0.0003, 0.0000) | **0.008** | 0.0036  (-0.0001, 0.0072) | 0.057 | 0.0002  (0.0000, 0.0003) | **0.033** | 0.0016  (-0.0021, 0.0053) | 0.390 | 0.0001  (-0.0001, 0.0002) | 0.406 |
|  | Riboflavin (mg) | ARIMA(3,0,0) errors | 0.941 | 0.2975  (0.2369, 0.3581) | **<0.001** | 0.0003  (-0.0006, 0.0012) | 0.528 | -0.1516  (-0.2383, -0.0649) | **0.001** | 0.0003  (-0.0014, 0.0020) | 0.738 | -0.0159  (-0.0978, 0.0661) | 0.704 | -0.0002  (-0.0020, 0.0015) | 0.806 |
|  | Niacin (mg) | ARIMA(2,0,1) errors | 0.793 | 2.2130  (2.1701, 2.2559) | **<0.001** | -0.0007  (-0.0014, -0.0001) | **0.023** | 0.0683  (0.0046, 0.1321) | **0.036** | 0.0015  (0.0003, 0.0028) | **0.016** | 0.0150  (-0.0487, 0.0788) | 0.644 | -0.0002  (-0.0014, 0.0010) | 0.749 |
|  | Dietary Folate Equivalent (mcg) | ARIMA(0,0,4) errors | 0.887 | 24.4763  (23.2472, 25.7053) | **<0.001** | -0.0217  (-0.0400, -0.0035) | **0.019** | 2.3982  (0.5971, 4.1993) | **0.009** | 0.0150  (-0.0202, 0.0502) | 0.404 | 1.1589  (-0.5998, 2.9176) | 0.197 | -0.0167  (-0.0522, 0.0187) | 0.355 |
|  | Vitamin B12 (mcg) | ARIMA(1,0,1) errors | 0.920 | 0.5562  (0.5371, 0.5754) | **<0.001** | -0.0003  (-0.0006, 0.0000) | 0.050 | 0.0247  (0.0007, 0.0487) | **0.044** | 0.0005  (-0.0001, 0.0010) | 0.111 | 0.0066  (-0.0186, 0.0319) | 0.607 | -0.0007  (-0.0012, -0.0001) | **0.014** |
|  | Vitamin C (mg) | ARIMA(5,0,0) errors | 0.766 | 6.6657  (6.3916, 6.9398) | **<0.001** | 0.0061  (0.0022, 0.0101) | **0.003** | 0.2350  (-0.1313, 0.6012) | 0.209 | -0.0121  (-0.0200, -0.0043) | **0.002** | 0.1312  (-0.2300, 0.4924) | 0.476 | -0.0042  (-0.0121, 0.0036) | 0.292 |
| Note: Mixed Dishes and Fast Foods/Starchy Roots and Tubers Transactions omitted in ITS analysis as data points limited in duration and coverage | | | | | | | | | | | | | | |  |
