# Supplementary material for: Food purchase patterns in Nairobi before, during, and after the COVID-19 pandemic lockdown measures
Source: PLOS Glob Public Health. 2026 Jun 1;6(6):e0006544. doi: 10.1371/journal.pgph.0006544 (PMC13225382; doi:10.1371/journal.pgph.0006544)
Supplement: S3 Table — (DOCX) [file pgph.0006544.s004.docx]

**S3 Table: Parameter estimates, confidence intervals, and Z-test p-values from the pre-pandemic ITS-GLS models predicting the weekly proportion of NOVA classification and weekly mean nutrient values per 100g/100ml of food**

| **Variable** | **Category** | **Optimal ITS -Generalised Least Squares model** | **Intercept (β_0_)** | | **Pre-COVID (β_1_)** | |
| --- | --- | --- | --- | --- | --- | --- |
|  |  |  | **Coefficient (95% CI)** | **Z test  p-value** | **Coefficient (95% CI)** | **Z test  p-value** |
| NOVA food classification | Processed Culinary Ingredients | corARMA(p=4, q=0) | 1.8757 (1.5725, 2.1789) | **<0.001** | -0.0044 (-0.0088, 0.0000) | **0.049** |
|  | Processed foods | corARMA(p=2, q=1) | 2.0365 (1.9198, 2.1532) | **<0.001** | 0.0019 (-0.0001, 0.0038) | 0.057 |
|  | Ultra-processed foods | corARMA(p=3, q=2) | 74.1370 (73.5444, 74.7296) | **<0.001** | 0.0372 (0.0273, 0.0470) | **<0.001** |
|  | Unprocessed/Minimally processed foods | corARMA(p=3, q=2) | 21.8161 (21.2357, 22.3966) | **<0.001** | -0.0317 (-0.0413, -0.0220) | **<0.001** |
| Proximates | Energy (kcal) | corARMA(p=4, q=4) | 541.0365 (519.8938, 562.1793) | **<0.001** | 0.4502 (0.0998, 0.8006) | **0.012** |
|  | Water (g) | corARMA(p=3, q=2) | 38.8811 (36.8126, 40.9495) | **<0.001** | -0.0057 (-0.0358, 0.0244) | 0.712 |
|  | Protein (g) | corARMA(p=3, q=2) | 6.2565 (6.1639, 6.3490) | **<0.001** | -0.0037 (-0.0051, -0.0023) | **<0.001** |
|  | Fat (g) | corARMA(p=3, q=3) | 11.3915 (10.8335, 11.9496) | **<0.001** | 0.0010 (-0.0070, 0.0091) | 0.803 |
|  | Carbohydrate available (g) | corARMA(p=0, q=3) | 43.2290 (42.2337, 44.2242) | **<0.001** | 0.0134 (-0.0014, 0.0283) | 0.075 |
|  | Fibre (g) | corARMA(p=1, q=0) | 4.1189 (3.9105, 4.3273) | **<0.001** | -0.0036 (-0.0067, -0.0005) | **0.023** |
|  | Cholesterol (mg) | corARMA(p=2, q=2) | 20.4780 (19.9633, 20.9926) | **<0.001** | 0.0138 (0.0052, 0.0223) | **0.002** |
| Minerals | Calcium (mg) | corARMA(p=4, q=4) | 97.0263 (94.0141, 100.0384) | **<0.001** | -0.0316 (-0.0765, 0.0132) | 0.167 |
|  | Iron (mg) | corARMA(p=1, q=0) | 2.2174 (2.1428, 2.2920) | **<0.001** | -0.0020 (-0.0031, -0.0009) | **0.001** |
|  | Magnesium (mg) | corARMA(p=1, q=2) | 34.7198 (32.7807, 36.6588) | **<0.001** | -0.0173 (-0.0449, 0.0104) | 0.221 |
|  | Phosphorus (mg) | corARMA(p=0, q=4) | 136.3028 (131.5035, 141.1021) | **<0.001** | -0.0158 (-0.0872, 0.0556) | 0.665 |
|  | Potassium (mg) | corARMA(p=1, q=0) | 307.4551 (299.4440, 315.4661) | **<0.001** | -0.1874 (-0.3069, -0.0680) | **0.002** |
|  | Sodium (mg) | corARMA(p=3, q=2) | 338.8226 (309.3716, 368.2735) | **<0.001** | -0.5935 (-1.0310, -0.1560) | **0.008** |
|  | Zinc (mg) | corARMA(p=1, q=0) | 0.7553 (0.7373, 0.7733) | **<0.001** | -0.0001 (-0.0004, 0.0001) | 0.327 |
|  | Selenium (mcg) | corARMA(p=1, q=0) | 6.6221 (6.5062, 6.7380) | **<0.001** | -0.0043 (-0.0061, -0.0026) | **<0.001** |
| Vitamins | Vitamin A-RE (mcg) | corARMA(p=3, q=1) | 161.1685 (144.4210, 177.9160) | **<0.001** | -0.3975 (-0.6413, -0.1538) | **0.001** |
|  | Thiamin (mg) | corARMA(p=1, q=0) | 0.1825 (0.1800, 0.1850) | **<0.001** | -0.0002 (-0.0002, -0.0001) | **<0.001** |
|  | Riboflavin (mg) | corARMA(p=4, q=3) | 0.2637 (0.2466, 0.2807) | **<0.001** | 0.0009 (0.0006, 0.0012) | **<0.001** |
|  | Niacin (mg) | corARMA(p=1, q=0) | 2.2087 (2.1711, 2.2463) | **<0.001** | -0.0006 (-0.0012, -0.0001) | **0.026** |
|  | Dietary Folate Equivalent (mcg) | corARMA(p=1, q=0) | 24.5634 (23.1758, 25.9510) | **<0.001** | -0.0236 (-0.0443, -0.0029) | **0.025** |
|  | Vitamin B12 (mcg) | corARMA(p=1, q=0) | 0.5560 (0.5352, 0.5769) | **<0.001** | -0.0003 (-0.0006, 0.0000) | 0.065 |
|  | Vitamin C (mg) | corARMA(p=1, q=1) | 6.6486 (6.2845, 7.0126) | **<0.001** | 0.0069 (0.0015, 0.0122) | **0.013** |
| Note: corARMA = accounts for autocorrelation; Mixed Dishes and Fast Foods/Starchy Roots and Tubers Transactions omitted in ITS analysis as data points limited in duration and coverage | | | | | | |
