# Supplementary material for: Food purchase patterns in Nairobi before, during, and after the COVID-19 pandemic lockdown measures
Source: PLOS Glob Public Health. 2026 Jun 1;6(6):e0006544. doi: 10.1371/journal.pgph.0006544 (PMC13225382; doi:10.1371/journal.pgph.0006544)
Supplement: S4 Table — (DOCX) [file pgph.0006544.s005.docx]

**S4 Table: Parameter estimates, confidence intervals, and Z-test p-values from the pre-pandemic ITS-ARIMA models predicting the weekly proportion of NOVA classification and weekly mean nutrient values per 100g/100ml of food**

| **Variable** | **Category** | **Optimal ITS- ARIMA model** | **Ljung-Box Pierce Test p-value** | **Intercept (β_0_)** | | **Pre-COVID (β_1_)** | |
| --- | --- | --- | --- | --- | --- | --- | --- |
|  |  |  |  | **Coefficient (95% CI)** | **Z test  p-value** | **Coefficient (95% CI)** | **Z test  p-value** |
| NOVA food classification | Processed Culinary Ingredients | ARIMA(3,0,2) errors | 0.680 | 1.8976 (1.6440, 2.1512) | **<0.001** | -0.0050 (-0.0396, 0.0296) | 0.778 |
|  | Processed foods | ARIMA(1,0,0) errors | 0.542 | 2.0582 (1.7805, 2.3359) | **<0.001** | 0.0017 (-0.0189, 0.0222) | 0.874 |
|  | Ultra-processed foods | ARIMA(0,0,3) errors | 0.812 | 74.2459 (72.7846, 75.7071) | **<0.001** | 0.0334 (0.0052, 0.0617) | **0.020** |
|  | Unprocessed/Minimally processed foods | ARIMA(0,0,3) errors | 0.998 | 21.7870 (20.4658, 23.1081) | **<0.001** | -0.0297 (-0.0563, -0.0030) | **0.029** |
| Proximates | Energy (kcal) | ARIMA(1,0,1) errors | 0.778 | 535.3615 (490.1861, 580.5369) | **<0.001** | 0.5579 (-0.1010, 1.2169) | 0.097 |
|  | Water (g) | ARIMA(3,0,2) errors | 0.977 | 38.8808 (36.8225, 40.9391) | **<0.001** | -0.0057 (-0.0356, 0.0243) | 0.710 |
|  | Protein (g) | ARIMA(1,0,0) errors | 0.798 | 6.2566 (6.1699, 6.3433) | **<0.001** | -0.0037 (-0.0050, -0.0024) | **<0.001** |
|  | Fat (g) | ARIMA(1,0,1) errors | 0.615 | 11.4135 (10.8794, 11.9476) | **<0.001** | 0.0006 (-0.0071, 0.0084) | 0.875 |
|  | Carbohydrate available (g) | ARIMA(2,0,0) errors | 0.908 | 43.2737 (42.0326, 44.5148) | **<0.001** | 0.0125 (-0.0059, 0.0309) | 0.184 |
|  | Fibre (g) | ARIMA(1,0,0) errors | 0.591 | 4.1189 (3.9117, 4.3260) | **<0.001** | -0.0036 (-0.0067, -0.0005) | **0.023** |
|  | Cholesterol (mg) | ARIMA(2,0,0) errors | 0.950 | 20.3423 (19.0723, 21.6124) | **<0.001** | 0.0179 (-0.0009, 0.0366) | 0.062 |
| Minerals | Calcium (mg) | ARIMA(0,0,5) errors | 0.851 | 97.0510 (93.8982, 100.2037) | **<0.001** | -0.0314 (-0.0785, 0.0156) | 0.191 |
|  | Iron (mg) | ARIMA(1,0,0) errors | 0.775 | 2.2174 (2.1434, 2.2914) | **<0.001** | -0.0020 (-0.0031, -0.0008) | **0.001** |
|  | Magnesium (mg) | ARIMA(1,0,2) errors | 0.889 | 34.7196 (32.7722, 36.6669) | **<0.001** | -0.0173 (-0.0456, 0.0111) | 0.233 |
|  | Phosphorus (mg) | ARIMA(0,0,5) errors | 0.996 | 136.4325 (132.6932, 140.1718) | **<0.001** | -0.0187 (-0.0746, 0.0371) | 0.511 |
|  | Potassium (mg) | ARIMA(1,0,0) errors | 0.986 | 307.4552 (299.5100, 315.4004) | **<0.001** | -0.1875 (-0.3061, -0.0688) | **0.002** |
|  | Sodium (mg) | ARIMA(1,0,0) errors | 0.880 | 339.1722 (312.4463, 365.8981) | **<0.001** | -0.6054 (-1.0034, -0.2075) | **0.003** |
|  | Zinc (mg) | ARIMA(1,0,0) errors | 0.924 | 0.7553 (0.7375, 0.7731) | **<0.001** | -0.0001 (-0.0005, 0.0002) | 0.416 |
|  | Selenium (mcg) | ARIMA(1,0,0) errors | 0.644 | 6.6221 (6.5071, 6.7372) | **<0.001** | -0.0043 (-0.0061, -0.0026) | **<0.001** |
| Vitamins | Vitamin A-RE (mcg) | ARIMA(3,0,1) errors | 0.843 | 161.1629 (140.2953, 182.0305) | **<0.001** | -0.3976 (-0.6967, -0.0984) | **0.009** |
|  | Thiamin (mg) | ARIMA(1,0,0) errors | 0.813 | 0.1825 (0.1800, 0.1850) | **<0.001** | -0.0002 (-0.0004, 0.0000) | 0.085 |
|  | Riboflavin (mg) | ARIMA(0,0,1) errors | 0.736 | 0.2887 (0.2366, 0.3409) | **<0.001** | 0.0005 (-0.0003, 0.0013) | 0.195 |
|  | Niacin (mg) | ARIMA(1,0,0) errors | 0.864 | 2.2087 (2.1714, 2.2460) | **<0.001** | -0.0006 (-0.0012, -0.0001) | **0.033** |
|  | Dietary Folate Equivalent (mcg) | ARIMA(1,0,0) errors | 0.700 | 24.5634 (23.1871, 25.9397) | **<0.001** | -0.0236 (-0.0441, -0.0031) | **0.024** |
|  | Vitamin B12 (mcg) | ARIMA(1,0,0) errors | 0.622 | 0.5560 (0.5353, 0.5768) | **<0.001** | -0.0003 (-0.0006, 0.0001) | 0.108 |
|  | Vitamin C (mg) | ARIMA(1,0,1) errors | 0.924 | 6.6483 (6.2847, 7.0120) | **<0.001** | 0.0069 (0.0014, 0.0123) | **0.013** |
| Note: Mixed Dishes and Fast Foods/Starchy Roots and Tubers Transactions omitted in ITS analysis as data points limited in duration and coverage | | | | | | | |
