# Supplementary material for: Food purchase patterns in Nairobi before, during, and after the COVID-19 pandemic lockdown measures
Source: PLOS Glob Public Health. 2026 Jun 1;6(6):e0006544. doi: 10.1371/journal.pgph.0006544 (PMC13225382; doi:10.1371/journal.pgph.0006544)
Supplement: S5 Table — (DOCX) [file pgph.0006544.s006.docx]

**S5 Table: Model evaluation metrics from the pre-pandemic ITS-GLS and ARIMA models predicting the weekly proportion of NOVA classification and the weekly mean nutrient values per 100g/100ml of food**

| **Variable** | **Category** | **Optimal ITS model** | **Train data**  **(1-115 weeks)** | | **Test data**  **(116-312 weeks)** | | | |
| --- | --- | --- | --- | --- | --- | --- | --- | --- |
|  |  |  | **AIC** | **BIC** | **RMSE** | **MAE** | **MAPE** | **MASE** |
| NOVA food classification | Processed Culinary Ingredients | generalised least squares via corARMA(p=4, q=0) | -1140.5267 | -1121.3121 | 0.0062 | 0.0058 | 72.6795 | 5.1483 |
|  |  | ARIMA(3,0,2) errors | -1140.1481 | -1118.1887 | 0.0072 | 0.0067 | 44.7261 | 0.4207 |
|  | Processed foods | generalised least squares via corARMA(p=2, q=1) | -1044.7060 | -1028.2364 | 0.0079 | 0.0061 | 25.5948 | 2.7092 |
|  |  | ARIMA(1,0,0) errors | -1044.1129 | -1033.1332 | 0.0079 | 0.0063 | 18.8814 | 0.2921 |
|  | Ultra-processed foods | generalised least squares via corARMA(p=3, q=2) | -652.7163 | -630.7568 | 0.0609 | 0.0558 | 6.7640 | 6.0490 |
|  |  | ARIMA(0,0,3) errors | -650.3444 | -633.8748 | 0.0540 | 0.0486 | 6.4162 | 0.0637 |
|  | Unprocessed/Minimally processed foods | generalised least squares via corARMA(p=3, q=2) | -672.2465 | -650.2871 | 0.0443 | 0.0392 | 27.3394 | 5.0189 |
|  |  | ARIMA(0,0,3) errors | -670.4588 | -653.9892 | 0.0404 | 0.0350 | 18.1188 | 0.1747 |
| Proximates | Carbohydrate available (g) | generalised least squares via corARMA(p=0, q=3) | 314.2214 | 330.6910 | 1.9961 | 1.6081 | 3.4792 | 2.8729 |
|  |  | ARIMA(2,0,0) errors | 314.2776 | 328.0023 | 1.8490 | 1.4356 | 3.2914 | 0.0326 |
|  | Cholesterol (mg) | generalised least squares via corARMA(p=2, q=2) | 316.1028 | 335.3173 | 1.8059 | 1.3259 | 5.6277 | 1.7415 |
|  |  | ARIMA(2,0,0) errors | 316.7513 | 330.4760 | 2.1730 | 1.7274 | 7.6764 | 0.0808 |
|  | Energy (kcal) | generalised least squares via corARMA(p=4, q=4) | 1078.5781 | 1108.7723 | 33.8724 | 27.1792 | 4.2554 | 1.5982 |
|  |  | ARIMA(1,0,1) errors | 1079.0047 | 1092.7294 | 41.4086 | 32.8071 | 5.1525 | 0.0575 |
|  | Fat (g) | generalised least squares via corARMA(p=3, q=3) | 86.5972 | 111.3015 | 0.8053 | 0.6838 | 5.8934 | 2.5519 |
|  |  | ARIMA(1,0,1) errors | 89.4427 | 103.1673 | 0.8530 | 0.7325 | 5.8558 | 0.0639 |
|  | Fibre (g) | generalised least squares via corARMA(p=1, q=0) | -14.4211 | -3.4414 | 0.8117 | 0.7412 | 22.3777 | 4.5322 |
|  |  | ARIMA(1,0,0) errors | -14.4211 | -3.4414 | 0.8082 | 0.7371 | 17.6847 | 0.1886 |
|  | Protein (g) | generalised least squares via corARMA(p=3, q=2) | -224.9781 | -203.0187 | 0.6741 | 0.6245 | 11.6086 | 8.1441 |
|  |  | ARIMA(1,0,0) errors | -224.6732 | -213.6934 | 0.6763 | 0.6257 | 10.2358 | 0.1036 |
|  | Water (g) | generalised least squares via corARMA(p=3, q=2) | 357.2838 | 379.2432 | 1.2934 | 1.0129 | 2.6872 | 1.2686 |
|  |  | ARIMA(3,0,2) errors | 357.2838 | 379.2432 | 1.2982 | 1.0125 | 2.7133 | 0.0263 |
| Minerals | Calcium (mg) | generalised least squares via corARMA(p=4, q=4) | 627.0759 | 657.2702 | 9.0981 | 6.6144 | 7.2852 | 2.1026 |
|  |  | ARIMA(0,0,5) errors | 627.1227 | 649.0822 | 8.9826 | 6.4770 | 6.3218 | 0.0681 |
|  | Iron (mg) | generalised least squares via corARMA(p=1, q=0) | -174.6086 | -163.6289 | 0.3650 | 0.3231 | 18.4193 | 3.6584 |
|  |  | ARIMA(1,0,0) errors | -174.6086 | -163.6289 | 0.3643 | 0.3222 | 14.9201 | 0.1533 |
|  | Magnesium (mg) | generalised least squares via corARMA(p=1, q=2) | 384.3256 | 400.7952 | 4.2398 | 3.9334 | 12.7627 | 3.9627 |
|  |  | ARIMA(1,0,2) errors | 384.3256 | 400.7952 | 4.1888 | 3.8710 | 10.9591 | 0.1151 |
|  | Phosphorus (mg) | generalised least squares via corARMA(p=0, q=4) | 732.3368 | 751.5513 | 11.5160 | 8.5451 | 6.4168 | 1.8790 |
|  |  | ARIMA(0,0,5) errors | 730.2970 | 752.2564 | 11.8262 | 8.9508 | 6.0743 | 0.0662 |
|  | Potassium (mg) | generalised least squares via corARMA(p=1, q=0) | 904.5141 | 915.4938 | 36.8529 | 34.7414 | 13.0313 | 4.0387 |
|  |  | ARIMA(1,0,0) errors | 904.5141 | 915.4938 | 36.7351 | 34.6098 | 11.3460 | 0.1168 |
|  | Selenium (mcg) | generalised least squares via corARMA(p=1, q=0) | -148.1574 | -137.1777 | 0.9845 | 0.8790 | 15.7828 | 6.7279 |
|  |  | ARIMA(1,0,0) errors | -148.1574 | -137.1777 | 0.9841 | 0.8777 | 13.1549 | 0.1379 |
|  | Sodium (mg) | generalised least squares via corARMA(p=3, q=2) | 1139.5848 | 1161.5442 | 83.6432 | 75.1795 | 38.3725 | 3.8729 |
|  |  | ARIMA(1,0,0) errors | 1145.0556 | 1156.0353 | 85.5053 | 76.9750 | 26.4979 | 0.2539 |
|  | Zinc (mg) | generalised least squares via corARMA(p=1, q=0) | -584.8230 | -573.8432 | 0.0676 | 0.0623 | 8.5844 | 4.2893 |
|  |  | ARIMA(1,0,0) errors | -584.8230 | -573.8432 | 0.0672 | 0.0619 | 7.7591 | 0.0829 |
| Vitamins | Dietary Folate Equivalent (mcg) | generalised least squares via corARMA(p=1, q=0) | 514.3203 | 525.3000 | 4.7029 | 4.3408 | 22.6447 | 4.1094 |
|  |  | ARIMA(1,0,0) errors | 514.3203 | 525.3000 | 4.6990 | 4.3360 | 17.9275 | 0.1869 |
|  | Niacin (mg) | generalised least squares via corARMA(p=1, q=0) | -306.4186 | -295.4389 | 0.2318 | 0.2051 | 9.9657 | 3.5701 |
|  |  | ARIMA(1,0,0) errors | -306.4186 | -295.4389 | 0.2314 | 0.2046 | 8.8310 | 0.0943 |
|  | Riboflavin (mg) | generalised least squares via corARMA(p=4, q=3) | -223.2571 | -195.8078 | 0.2549 | 0.2455 | 53.0627 | 9.4048 |
|  |  | ARIMA(0,0,1) errors | -219.0621 | -208.0824 | 0.1949 | 0.1870 | 97.2093 | 0.5826 |
|  | Thiamin (mg) | generalised least squares via corARMA(p=1, q=0) | -983.4128 | -972.4330 | 0.0235 | 0.0205 | 14.5058 | 8.7031 |
|  |  | ARIMA(1,0,0) errors | -983.4128 | -972.4330 | 0.0235 | 0.0205 | 12.1403 | 0.1185 |
|  | Vitamin A-RE (mcg) | generalised least squares via corARMA(p=3, q=1) | 972.8633 | 992.0778 | 48.5985 | 43.9965 | 72.6217 | 5.4112 |
|  |  | ARIMA(3,0,1) errors | 972.8633 | 992.0778 | 48.5462 | 43.8544 | 36.7088 | 0.3141 |
|  | Vitamin B12 (mcg) | generalised least squares via corARMA(p=1, q=0) | -609.1851 | -598.2054 | 0.0539 | 0.0485 | 9.8533 | 3.7176 |
|  |  | ARIMA(1,0,0) errors | -609.1851 | -598.2054 | 0.0537 | 0.0483 | 8.7665 | 0.0895 |
|  | Vitamin C (mg) | generalised least squares via corARMA(p=1, q=1) | 133.2658 | 146.9904 | 1.4545 | 1.2106 | 14.5087 | 5.5811 |
|  |  | ARIMA(1,0,1) errors | 133.2658 | 146.9905 | 1.4529 | 1.2084 | 18.3307 | 0.1717 |
| Note: AIC = Akaike Information Criterion; BIC = Bayesian Information Criterion; RMSE = Root Mean Squared Error; MAE = Mean Absolute Error; MAPE = Mean Absolute Percentage Error; MASE = Mean Absolute Scaled Error | | | | | | | | |
