# Supplementary material for: Food purchase patterns in Nairobi before, during, and after the COVID-19 pandemic lockdown measures
Source: PLOS Glob Public Health. 2026 Jun 1;6(6):e0006544. doi: 10.1371/journal.pgph.0006544 (PMC13225382; doi:10.1371/journal.pgph.0006544)
Supplement: S6 Table — (DOCX) [file pgph.0006544.s007.docx]

**S6 Table: Parameter estimates, confidence intervals, and Z-test p-values from the combined pre-pandemic and pandemic ITS-GLS models predicting the weekly proportion of NOVA classification and weekly mean nutrient values per 100g/100ml of food**

| **Variable** | **Category** | **Optimal ITS -Generalised Least Squares model** | **Intercept (β_0_)** | | **Pre-COVID (β_1_)** | | **Start of Lockdown (β_2_)** | | **COVID Period (β_3_)** | |
| --- | --- | --- | --- | --- | --- | --- | --- | --- | --- | --- |
|  |  |  | **Coefficient (95% CI)** | **Z test  p-value** | **Coefficient (95% CI)** | **Z test  p-value** | **Coefficient (95% CI)** | **Z test  p-value** | **Coefficient (95% CI)** | **Z test  P-value** |
| NOVA food classification | Processed Culinary Ingredients | corARMA(p=4, q=0) | 1.8793 (1.6399, 2.1187) | **<0.001** | -0.0045 (-0.0079, -0.0012) | **0.008** | 0.1997 (-0.0332, 0.4326) | 0.093 | 0.0034 (-0.0035, 0.0103) | 0.339 |
|  | Processed foods | corARMA(p=3, q=1) | 1.9582 (1.8183, 2.0981) | **<0.001** | 0.0036 (0.0013, 0.0058) | **0.002** | 0.4202 (0.1390, 0.7015) | **0.003** | 0.0009 (-0.0036, 0.0055) | 0.683 |
|  | Ultra-processed foods | corARMA(p=0, q=3) | 74.3079 (72.8367, 75.7790) | **<0.001** | 0.0318 (0.0106, 0.0530) | **0.003** | -2.4205 (-4.2994, -0.5416) | **0.012** | -0.0237 (-0.0666, 0.0192) | 0.279 |
|  | Unprocessed/Minimally processed foods | corARMA(p=0, q=3) | 21.7839 (20.4818, 23.0859) | **<0.001** | -0.0297 (-0.0485, -0.0109) | **0.002** | 1.6621 (0.0050, 3.3191) | **0.049** | 0.0199 (-0.0181, 0.0578) | 0.305 |
| Proximates | Energy (kcal) | corARMA(p=2, q=3) | 527.9135 (499.2293, 556.5976) | **<0.001** | 0.7323 (0.2760, 1.1885) | **0.002** | -3.7970 (-43.7781, 36.1842) | 0.852 | -0.1637 (-1.2494, 0.9221) | 0.768 |
|  | Water (g) | corARMA(p=2, q=4) | 38.8395 (37.2896, 40.3894) | **<0.001** | -0.0056 (-0.0274, 0.0162) | 0.613 | -1.1274 (-2.7374, 0.4826) | 0.170 | 0.0141 (-0.0312, 0.0594) | 0.542 |
|  | Protein (g) | corARMA(p=1, q=0) | 6.2551 (6.1758, 6.3344) | **<0.001** | -0.0037 (-0.0049, -0.0025) | **<0.001** | 0.2693 (0.1589, 0.3797) | **<0.001** | 0.0026 (0.0003, 0.0049) | **0.030** |
|  | Fat (g) | corARMA(p=3, q=2) | 11.5429 (11.4510, 11.6348) | **<0.001** | -0.0019 (-0.0034, -0.0004) | **0.013** | 0.7817 (0.5636, 0.9998) | **<0.001** | 0.0061 (0.0030, 0.0092) | **<0.001** |
|  | Carbohydrate available (g) | corARMA(p=3, q=2) | 43.2617 (42.0306, 44.4927) | **<0.001** | 0.0123 (-0.0051, 0.0296) | 0.167 | -0.4142 (-1.7445, 0.9160) | 0.542 | -0.0137 (-0.0497, 0.0222) | 0.455 |
|  | Fibre (g) | corARMA(p=4, q=2) | 4.0838 (3.8588, 4.3088) | **<0.001** | -0.0027 (-0.0060, 0.0005) | 0.099 | 0.4393 (0.1526, 0.7260) | **0.003** | 0.0014 (-0.0051, 0.0080) | 0.669 |
|  | Cholesterol (mg) | corARMA(p=3, q=1) | 20.4020 (19.7648, 21.0391) | **<0.001** | 0.0160 (0.0058, 0.0262) | **0.002** | 1.0754 (-0.1245, 2.2753) | 0.079 | -0.0363 (-0.0570, -0.0155) | **0.001** |
| Minerals | Calcium (mg) | corARMA(p=4, q=1) | 96.9213 (95.6865, 98.1561) | **<0.001** | -0.0256 (-0.0456, -0.0056) | **0.012** | 11.0197 (8.4428, 13.5966) | **<0.001** | -0.0885 (-0.1281, -0.0490) | **<0.001** |
|  | Iron (mg) | corARMA(p=4, q=3) | 2.1995 (2.1618, 2.2373) | **<0.001** | -0.0017 (-0.0023, -0.0011) | **<0.001** | 0.1433 (0.0669, 0.2198) | **<0.001** | 0.0010 (-0.0002, 0.0022) | 0.094 |
|  | Magnesium (mg) | corARMA(p=3, q=2) | 34.7909 (33.4566, 36.1251) | **<0.001** | -0.0207 (-0.0398, -0.0015) | **0.034** | 3.5064 (1.9055, 5.1073) | **<0.001** | -0.0001 (-0.0397, 0.0395) | 0.995 |
|  | Phosphorus (mg) | corARMA(p=0, q=4) | 135.7946 (130.6777, 140.9115) | **<0.001** | -0.0020 (-0.0772, 0.0732) | 0.959 | 11.7519 (4.5644, 18.9394) | **0.001** | -0.0921 (-0.2410, 0.0568) | 0.225 |
|  | Potassium (mg) | corARMA(p=4, q=0) | 307.1425 (296.8701, 317.4149) | **<0.001** | -0.1756 (-0.3250, -0.0262) | **0.021** | 29.7621 (16.1914, 43.3327) | **<0.001** | 0.0261 (-0.2724, 0.3246) | 0.864 |
|  | Sodium (mg) | corARMA(p=4, q=3) | 338.6712 (311.3657, 365.9766) | **<0.001** | -0.5706 (-0.9620, -0.1791) | **0.004** | 56.4559 (23.3899, 89.5218) | **0.001** | -0.3477 (-1.1430, 0.4477) | 0.392 |
|  | Zinc (mg) | corARMA(p=1, q=0) | 0.7539 (0.7356, 0.7722) | **<0.001** | -0.0001 (-0.0004, 0.0002) | 0.481 | 0.0431 (0.0182, 0.0679) | **0.001** | 0.0000 (-0.0005, 0.0006) | 0.926 |
|  | Selenium (mcg) | corARMA(p=3, q=2) | 6.5973 (6.5458, 6.6488) | **<0.001** | -0.0041 (-0.0049, -0.0033) | **<0.001** | 0.2007 (0.0955, 0.3058) | **<0.001** | 0.0049 (0.0033, 0.0066) | **<0.001** |
| Vitamins | Vitamin A-RE (mcg) | corARMA(p=4, q=0) | 162.9882 (150.2623, 175.7141) | **<0.001** | -0.4186 (-0.6026, -0.2346) | **<0.001** | 16.1563 (-0.0380, 32.3507) | 0.051 | 0.2358 (-0.1338, 0.6053) | 0.211 |
|  | Thiamin (mg) | corARMA(p=1, q=0) | 0.1827 (0.1800, 0.1853) | **<0.001** | -0.0002 (-0.0002, -0.0001) | **<0.001** | 0.0034 (-0.0003, 0.0071) | 0.074 | 0.0002 (0.0001, 0.0002) | **<0.001** |
|  | Riboflavin (mg) | corARMA(p=4, q=3) | 0.2635 (0.2502, 0.2768) | **<0.001** | 0.0009 (0.0007, 0.0012) | **<0.001** | -0.2351 (-0.2626, -0.2075) | **<0.001** | 0.0004 (0.0000, 0.0008) | 0.064 |
|  | Niacin (mg) | corARMA(p=4, q=3) | 2.2138 (2.1596, 2.2681) | **<0.001** | -0.0008 (-0.0015, 0.0000) | 0.050 | 0.0815 (0.0211, 0.1419) | **0.008** | 0.0013 (-0.0003, 0.0029) | 0.115 |
|  | Dietary Folate Equivalent (mcg) | corARMA(p=1, q=0) | 24.5575 (23.2270, 25.8880) | **<0.001** | -0.0236 (-0.0433, -0.0039) | **0.019** | 2.7273 (0.7742, 4.6805) | **0.006** | 0.0133 (-0.0253, 0.0520) | 0.499 |
|  | Vitamin B12 (mcg) | corARMA(p=4, q=2) | 0.5550 (0.5457, 0.5644) | **<0.001** | -0.0003 (-0.0005, -0.0002) | **<0.001** | 0.0305 (0.0125, 0.0485) | **0.001** | 0.0003 (0.0000, 0.0006) | **0.025** |
|  | Vitamin C (mg) | corARMA(p=1, q=1) | 6.6832 (6.3794, 6.9869) | **<0.001** | 0.0059 (0.0015, 0.0104) | **0.009** | 0.2500 (-0.1634, 0.6634) | 0.236 | -0.0123 (-0.0212, -0.0035) | **0.006** |
| Note: corARMA = accounts for autocorrelation; Mixed Dishes and Fast Foods/Starchy Roots and Tubers FTransactions omitted in ITS analysis as data points limited in duration and coverage | | | | | | | | | | |
