# Supplementary material for: Food purchase patterns in Nairobi before, during, and after the COVID-19 pandemic lockdown measures
Source: PLOS Glob Public Health. 2026 Jun 1;6(6):e0006544. doi: 10.1371/journal.pgph.0006544 (PMC13225382; doi:10.1371/journal.pgph.0006544)
Supplement: S7 Table — (DOCX) [file pgph.0006544.s008.docx]

**S7 Table: Parameter estimates, confidence intervals, and Z-test p-values from the combined pre-pandemic and pandemic ITS-ARIMA models predicting the weekly proportion of NOVA classification and weekly mean nutrient values per 100g/100ml of food**

| **Variable** | **Category** | **Optimal ITS- ARIMA model** | **Ljung-Box Pierce Test p-value** | **Intercept (β_0_)** | | **Pre-COVID (β_1_)** | | **Start of Lockdown (β_2_)** | | **COVID Period (β_3_)** | |
| --- | --- | --- | --- | --- | --- | --- | --- | --- | --- | --- | --- |
|  |  |  |  | **Coefficient (95% CI)** | **Z test  p-value** | **Coefficient (95% CI)** | **Z test  p-value** | **Coefficient (95% CI)** | **Z test  p-value** | **Coefficient (95% CI)** | **Z test  P-value** |
| NOVA food classification | Processed Culinary Ingredients | ARIMA(5,0,0) errors | 0.976 | 1.8795 (1.6391, 2.1199) | **<0.001** | -0.0045 (-0.0232, 0.0141) | 0.635 | 0.1988 (-0.0667, 0.4643) | 0.142 | 0.0034 (-0.0123, 0.0191) | 0.672 |
|  | Processed foods | ARIMA(1,0,0) errors | 0.912 | 1.9958 (1.6134, 2.3782) | **<0.001** | 0.0034 (-0.0125, 0.0192) | 0.677 | 0.3947 (-0.0900, 0.8794) | 0.110 | 0.0012 (-0.0163, 0.0186) | 0.897 |
|  | Ultra-processed foods | ARIMA(0,0,3) errors | 0.661 | 74.3077 (72.8511, 75.7643) | **<0.001** | 0.0318 (0.0064, 0.0572) | **0.014** | -2.4205 (-4.2897, -0.5512) | **0.011** | -0.0237 (-0.0683, 0.0209) | 0.298 |
|  | Unprocessed/Minimally processed foods | ARIMA(0,0,3) errors | 0.948 | 21.7837 (20.4941, 23.0732) | **<0.001** | -0.0297 (-0.0532, -0.0063) | **0.013** | 1.6648 (0.0010, 3.3285) | **0.050** | 0.0198 (-0.0202, 0.0599) | 0.331 |
| Proximates | Energy (kcal) | ARIMA(1,0,1) errors | 0.806 | 531.7861 (497.8225, 565.7496) | **<0.001** | 0.6735 (0.1889, 1.1580) | **0.006** | 1.1123 (-37.9800, 40.2046) | 0.956 | -0.1222 (-1.1083, 0.8639) | 0.808 |
|  | Water (g) | ARIMA(5,0,0) errors | 0.925 | 39.0933 (37.2795, 40.9072) | **<0.001** | -0.0096 (-0.0349, 0.0157) | 0.457 | -0.8376 (-2.5789, 0.9038) | 0.346 | 0.0178 (-0.0346, 0.0703) | 0.505 |
|  | Protein (g) | ARIMA(1,0,0) errors | 0.437 | 6.2551 (6.1766, 6.3336) | **<0.001** | -0.0037 (-0.0049, -0.0025) | **<0.001** | 0.2693 (0.1587, 0.3799) | **<0.001** | 0.0026 (0.0003, 0.0048) | **0.028** |
|  | Fat (g) | ARIMA(1,0,2) errors | 0.972 | 11.3908 (10.9407, 11.8409) | **<0.001** | 0.0015 (-0.0050, 0.0080) | 0.646 | 0.2606 (-0.2670, 0.7882) | 0.333 | 0.0080 (-0.0048, 0.0208) | 0.220 |
|  | Carbohydrate available (g) | ARIMA(3,0,2) errors | 0.760 | 43.2617 (42.0411, 44.4823) | **<0.001** | 0.0123 (-0.0050, 0.0296) | 0.165 | -0.4142 (-1.7722, 0.9439) | 0.550 | -0.0137 (-0.0493, 0.0219) | 0.451 |
|  | Fibre (g) | ARIMA(1,0,0) errors | 0.544 | 4.0935 (3.8535, 4.3335) | **<0.001** | -0.0029 (-0.0065, 0.0006) | 0.103 | 0.4683 (0.1384, 0.7983) | **0.005** | 0.0013 (-0.0056, 0.0083) | 0.706 |
|  | Cholesterol (mg) | ARIMA(3,0,2) errors | 0.816 | 20.4283 (19.2134, 21.6432) | **<0.001** | 0.0160 (-0.0013, 0.0333) | 0.070 | 1.0844 (-0.3302, 2.4989) | 0.133 | -0.0354 (-0.0708, 0.0000) | 0.050 |
| Minerals | Calcium (mg) | ARIMA(0,0,4) errors | 0.892 | 96.3079 (92.2823, 100.3336) | **<0.001** | -0.0113 (-0.0711, 0.0485) | 0.712 | 9.1880 (3.2441, 15.1319) | **0.002** | -0.0919 (-0.2086, 0.0249) | 0.123 |
|  | Iron (mg) | ARIMA(1,0,0) errors | 0.977 | 2.2170 (2.1348, 2.2992) | **<0.001** | -0.0020 (-0.0032, -0.0007) | **0.002** | 0.1642 (0.0446, 0.2839) | **0.007** | 0.0010 (-0.0014, 0.0033) | 0.434 |
|  | Magnesium (mg) | ARIMA(1,0,4) errors | 0.970 | 34.8030 (33.1738, 36.4322) | **<0.001** | -0.0194 (-0.0425, 0.0038) | 0.101 | 3.2042 (1.4083, 5.0001) | **<0.001** | 0.0008 (-0.0467, 0.0482) | 0.975 |
|  | Phosphorus (mg) | ARIMA(0,0,4) errors | 0.879 | 135.7940 (130.7080, 140.8801) | **<0.001** | -0.0020 (-0.0772, 0.0732) | 0.959 | 11.7521 (4.3970, 19.1073) | **0.002** | -0.0921 (-0.2397, 0.0555) | 0.221 |
|  | Potassium (mg) | ARIMA(0,0,5) errors | 0.985 | 307.0053 (298.5408, 315.4698) | **<0.001** | -0.1740 (-0.2996, -0.0484) | **0.007** | 29.2325 (16.7907, 41.6744) | **<0.001** | 0.0389 (-0.2067, 0.2844) | 0.756 |
|  | Sodium (mg) | ARIMA(4,0,1) errors | 0.807 | 339.7192 (310.0142, 369.4243) | **<0.001** | -0.5927 (-1.0252, -0.1602) | **0.007** | 59.6368 (19.5862, 99.6874) | **0.004** | -0.3473 (-1.2156, 0.5210) | 0.433 |
|  | Zinc (mg) | ARIMA(1,0,0) errors | 0.977 | 0.7539 (0.7357, 0.7721) | **<0.001** | -0.0001 (-0.0004, 0.0002) | 0.535 | 0.0430 (0.0173, 0.0688) | **0.001** | 0.0000 (-0.0005, 0.0006) | 0.928 |
|  | Selenium (mcg) | ARIMA(1,0,0) errors | 0.785 | 6.6238 (6.5166, 6.7310) | **<0.001** | -0.0044 (-0.0060, -0.0029) | **<0.001** | 0.2052 (0.0523, 0.3581) | **0.009** | 0.0056 (0.0025, 0.0087) | **<0.001** |
| Vitamins | Vitamin A-RE (mcg) | ARIMA(4,0,0) errors | 0.856 | 162.9897 (148.9476, 177.0318) | **<0.001** | -0.4186 (-0.6216, -0.2156) | **<0.001** | 16.1529 (-0.5376, 32.8433) | 0.058 | 0.2356 (-0.1430, 0.6142) | 0.223 |
|  | Thiamin (mg) | ARIMA(3,0,0) errors | 0.956 | 0.1827 (0.1801, 0.1853) | **<0.001** | -0.0002 (-0.0003, 0.0000) | **0.024** | 0.0034 (-0.0003, 0.0070) | 0.070 | 0.0002 (0.0000, 0.0003) | **0.046** |
|  | Riboflavin (mg) | ARIMA(0,0,1) errors | 0.676 | 0.2906 (0.2501, 0.3312) | **<0.001** | 0.0005 (-0.0001, 0.0011) | 0.128 | -0.1872 (-0.2484, -0.1260) | **<0.001** | 0.0005 (-0.0007, 0.0016) | 0.444 |
|  | Niacin (mg) | ARIMA(1,0,2) errors | 0.993 | 2.2139 (2.1542, 2.2736) | **<0.001** | -0.0008 (-0.0016, 0.0001) | 0.087 | 0.0732 (-0.0039, 0.1502) | 0.063 | 0.0015 (-0.0002, 0.0033) | 0.087 |
|  | Dietary Folate Equivalent (mcg) | ARIMA(1,0,0) errors | 0.716 | 24.5575 (23.2400, 25.8749) | **<0.001** | -0.0236 (-0.0431, -0.0041) | **0.018** | 2.7274 (0.7941, 4.6607) | **0.006** | 0.0133 (-0.0249, 0.0516) | 0.495 |
|  | Vitamin B12 (mcg) | ARIMA(1,0,0) errors | 0.518 | 0.5560 (0.5364, 0.5757) | **<0.001** | -0.0003 (-0.0006, 0.0000) | 0.072 | 0.0287 (0.0045, 0.0529) | **0.020** | 0.0003 (-0.0003, 0.0008) | 0.401 |
|  | Vitamin C (mg) | ARIMA(5,0,0) errors | 0.784 | 6.6647 (6.3341, 6.9952) | **<0.001** | 0.0061 (0.0013, 0.0109) | **0.012** | 0.2674 (-0.1638, 0.6986) | 0.224 | -0.0130 (-0.0227, -0.0033) | **0.009** |
| Note: Mixed Dishes and Fast Foods/Starchy Roots and Tubers Food group Transactions omitted in ITS analysis as data points limited in duration and coverage | | | | | | | | | | | |
