# Supplementary material for: Food purchase patterns in Nairobi before, during, and after the COVID-19 pandemic lockdown measures
Source: PLOS Glob Public Health. 2026 Jun 1;6(6):e0006544. doi: 10.1371/journal.pgph.0006544 (PMC13225382; doi:10.1371/journal.pgph.0006544)
Supplement: S8 Table — (DOCX) [file pgph.0006544.s009.docx]

**S8 Table: Model evaluation metrics from the combined pre-pandemic and pandemic ITS-GLS and ARIMA models predicting the weekly proportion of NOVA classification, and the weekly mean nutrient values per 100g/100ml of food**

| **Variable** | **Category** | **Optimal ITS model** | **Train data**  **(1-197 weeks)** | | **Test data**  **(198-312 weeks)** | | | |
| --- | --- | --- | --- | --- | --- | --- | --- | --- |
|  |  |  | **AIC** | **BIC** | **RMSE** | **MAE** | **MAPE** | **MASE** |
| NOVA food classification | Processed Culinary Ingredients | generalised least squares corARMA(p=4, q=0) | -1993.9190 | -1964.3702 | 0.0016 | 0.0012 | 8.7764 | 1.0706 |
|  |  | ARIMA(5,0,0) errors | -1991.9412 | -1959.1092 | 0.0016 | 0.0012 | 7.8175 | 0.0768 |
|  | Processed foods | generalised least squares corARMA(p=3, q=1) | -1767.6466 | -1738.0977 | 0.0071 | 0.0060 | 17.1945 | 2.7277 |
|  |  | ARIMA(1,0,0) errors | -1763.0084 | -1743.3092 | 0.0071 | 0.0060 | 21.2409 | 0.2375 |
|  | Ultra-processed foods | generalised least squares corARMA(p=0, q=3) | -1115.9419 | -1089.6763 | 0.0197 | 0.0131 | 1.7123 | 1.4770 |
|  |  | ARIMA(0,0,3) errors | -1115.9419 | -1089.6763 | 0.0197 | 0.0132 | 1.7427 | 0.0173 |
|  | Unprocessed/Minimally processed foods | generalised least squares corARMA(p=0, q=3) | -1162.9353 | -1136.6697 | 0.0163 | 0.0109 | 5.8096 | 1.4805 |
|  |  | ARIMA(0,0,3) errors | -1162.9353 | -1136.6697 | 0.0163 | 0.0109 | 5.6677 | 0.0546 |
| Proximates | Carbohydrate available (g) | generalised least squares corARMA(p=3, q=2) | 513.5080 | 546.3400 | 1.4278 | 1.1736 | 2.6648 | 2.2431 |
|  |  | ARIMA(3,0,2) errors | 513.5080 | 546.3400 | 1.4267 | 1.1663 | 2.6040 | 0.0265 |
|  | Cholesterol (mg) | generalised least squares corARMA(p=3, q=1) | 531.3810 | 560.9298 | 3.2754 | 2.8719 | 14.0614 | 3.4703 |
|  |  | ARIMA(3,0,2) errors | 536.6796 | 569.5116 | 3.1383 | 2.7157 | 11.2706 | 0.1242 |
|  | Energy (kcal) | generalised least squares corARMA(p=2, q=3) | 1827.0280 | 1859.8600 | 56.8611 | 49.0088 | 7.0377 | 3.0578 |
|  |  | ARIMA(1,0,1) errors | 1827.7753 | 1850.7577 | 56.0630 | 48.0544 | 7.6059 | 0.0804 |
|  | Fat (g) | generalised least squares corARMA(p=3, q=2) | 154.1546 | 186.9866 | 0.7365 | 0.5698 | 4.4540 | 2.2432 |
|  |  | ARIMA(1,0,2) errors | 168.4710 | 194.7366 | 1.1645 | 0.9526 | 7.9594 | 0.0807 |
|  | Fibre (g) | generalised least squares corARMA(p=4, q=2) | -10.7869 | 25.3284 | 0.2963 | 0.1709 | 4.2411 | 1.0782 |
|  |  | ARIMA(1,0,0) errors | -8.3029 | 11.3963 | 0.2964 | 0.1607 | 3.7053 | 0.0399 |
|  | Protein (g) | generalised least squares corARMA(p=1, q=0) | -382.9099 | -363.2107 | 0.2011 | 0.1749 | 2.9488 | 2.3423 |
|  |  | ARIMA(1,0,0) errors | -382.9099 | -363.2107 | 0.2011 | 0.1749 | 2.8394 | 0.0289 |
|  | Water (g) | generalised least squares corARMA(p=2, q=4) | 591.5225 | 627.6378 | 1.5719 | 1.2996 | 3.3939 | 1.7045 |
|  |  | ARIMA(5,0,0) errors | 597.1207 | 629.9527 | 1.6032 | 1.3315 | 3.6029 | 0.0350 |
| Minerals | Calcium (mg) | generalised least squares corARMA(p=4, q=1) | 1159.9099 | 1192.7420 | 7.4552 | 5.5090 | 6.2792 | 2.0875 |
|  |  | ARIMA(0,0,4) errors | 1163.1499 | 1192.6987 | 6.8610 | 4.8450 | 4.9582 | 0.0497 |
|  | Iron (mg) | generalised least squares corARMA(p=4, q=3) | -298.4026 | -259.0042 | 0.1486 | 0.0950 | 4.6506 | 1.0859 |
|  |  | ARIMA(1,0,0) errors | -291.9841 | -272.2848 | 0.1667 | 0.1145 | 5.1368 | 0.0543 |
|  | Magnesium (mg) | generalised least squares corARMA(p=3, q=2) | 656.1664 | 688.9984 | 2.3105 | 1.8789 | 5.7201 | 1.8803 |
|  |  | ARIMA(1,0,4) errors | 657.7155 | 690.5475 | 2.1770 | 1.7307 | 4.8532 | 0.0505 |
|  | Phosphorus (mg) | generalised least squares corARMA(p=0, q=4) | 1317.7073 | 1347.2561 | 8.2295 | 5.7112 | 4.2644 | 1.5552 |
|  |  | ARIMA(0,0,4) errors | 1317.7073 | 1347.2561 | 8.2330 | 5.7126 | 3.9434 | 0.0411 |
|  | Potassium (mg) | generalised least squares corARMA(p=4, q=0) | 1530.2624 | 1559.8112 | 12.1301 | 8.5952 | 2.9044 | 1.0245 |
|  |  | ARIMA(0,0,5) errors | 1531.3739 | 1564.2059 | 12.2064 | 8.9109 | 2.9892 | 0.0294 |
|  | Selenium (mcg) | generalised least squares corARMA(p=3, q=2) | -221.0491 | -188.2171 | 0.3244 | 0.2851 | 4.4239 | 2.1123 |
|  |  | ARIMA(1,0,0) errors | -220.8382 | -201.1390 | 0.2922 | 0.2507 | 3.6889 | 0.0394 |
|  | Sodium (mg) | generalised least squares corARMA(p=4, q=3) | 1909.4987 | 1948.8971 | 87.7782 | 80.9773 | 43.4376 | 4.6522 |
|  |  | ARIMA(4,0,1) errors | 1923.1703 | 1956.0023 | 89.1696 | 82.0696 | 28.8906 | 0.2743 |
|  | Zinc (mg) | generalised least squares corARMA(p=1, q=0) | -992.5608 | -972.8616 | 0.0285 | 0.0175 | 2.2592 | 1.1781 |
|  |  | ARIMA(1,0,0) errors | -992.5608 | -972.8616 | 0.0286 | 0.0175 | 2.1389 | 0.0230 |
| Vitamins | Dietary Folate Equivalent (mcg) | generalised least squares corARMA(p=1, q=0) | 824.5501 | 844.2493 | 1.5375 | 1.0473 | 4.5216 | 1.0151 |
|  |  | ARIMA(1,0,0) errors | 824.5501 | 844.2493 | 1.5456 | 1.0586 | 4.2986 | 0.0449 |
|  | Niacin (mg) | generalised least squares corARMA(p=4, q=3) | -510.2907 | -470.8923 | 0.0802 | 0.0651 | 2.8626 | 1.2077 |
|  |  | ARIMA(1,0,2) errors | -509.5038 | -483.2381 | 0.0738 | 0.0593 | 2.5848 | 0.0270 |
|  | Riboflavin (mg) | generalised least squares corARMA(p=4, q=3) | -483.6306 | -444.2322 | 0.1252 | 0.1102 | 32.9738 | 3.3692 |
|  |  | ARIMA(0,0,1) errors | -471.5330 | -451.8338 | 0.0998 | 0.0851 | 42.1963 | 0.3169 |
|  | Thiamin (mg) | generalised least squares corARMA(p=1, q=0) | -1720.4605 | -1700.7613 | 0.0057 | 0.0047 | 2.8502 | 1.7774 |
|  |  | ARIMA(3,0,0) errors | -1716.7687 | -1690.5031 | 0.0057 | 0.0047 | 2.7360 | 0.0275 |
|  | Vitamin A-RE (mcg) | generalised least squares corARMA(p=4, q=0) | 1598.1883 | 1627.7372 | 15.2654 | 12.3675 | 11.9710 | 1.5170 |
|  |  | ARIMA(4,0,0) errors | 1598.1883 | 1627.7372 | 15.3026 | 12.4098 | 10.2337 | 0.0931 |
|  | Vitamin B12 (mcg) | generalised least squares corARMA(p=4, q=2) | -1062.5569 | -1026.4417 | 0.0365 | 0.0307 | 5.5076 | 2.2692 |
|  |  | ARIMA(1,0,0) errors | -1059.2586 | -1039.5594 | 0.0296 | 0.0244 | 4.5804 | 0.0449 |
|  | Vitamin C (mg) | generalised least squares corARMA(p=1, q=1) | 158.3983 | 181.3807 | 0.2969 | 0.2413 | 3.6177 | 1.0662 |
|  |  | ARIMA(5,0,0) errors | 160.3240 | 193.1561 | 0.2861 | 0.2308 | 3.4897 | 0.0322 |
| Note: AIC = Akaike Information Criterion; BIC = Bayesian Information Criterion; RMSE = Root Mean Squared Error; MAE = Mean Absolute Error; MAPE = Mean Absolute Percentage Error; MASE = Mean Absolute Scaled Error | | | | | | | | |
