# Supplementary figures and images for: Food purchase patterns in Nairobi before, during, and after the COVID-19 pandemic lockdown measures
Source: PLOS Glob Public Health. 2026 Jun 1;6(6):e0006544. doi: 10.1371/journal.pgph.0006544 (PMC13225382; doi:10.1371/journal.pgph.0006544)

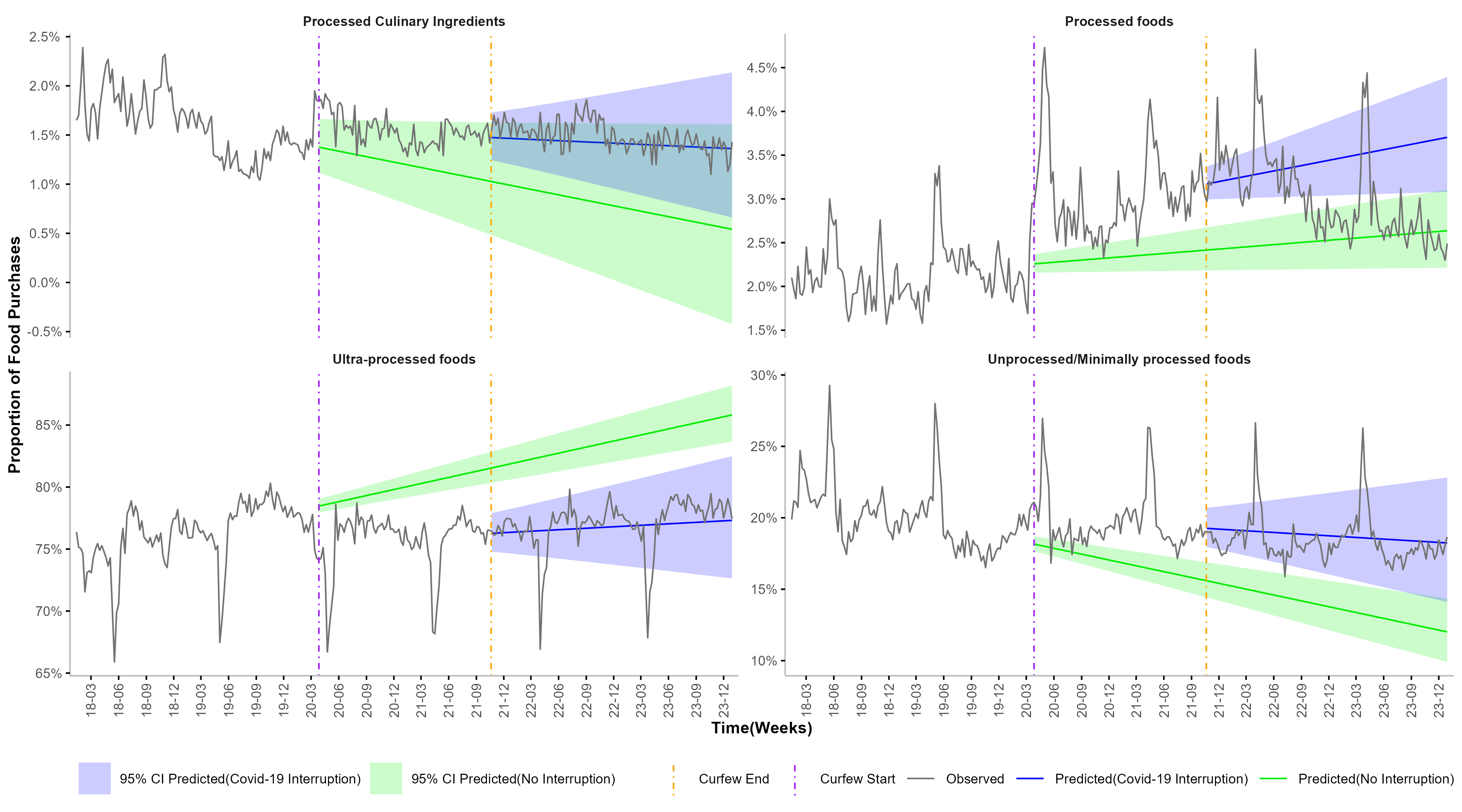

Supplement: S1 Fig — Curfew start vertical line = 27th March 2020 (start of pandemic restrictions). Curfew end vertical line = 20th October 2021 (end of pandemic restrictions). Predictions were estimated in two parts: by extrapolating the pre-pandemic trend and by extrapolating the combined pre-pandemic and pandemic trend. (TIFF) [file pgph.0006544.s010.tiff]

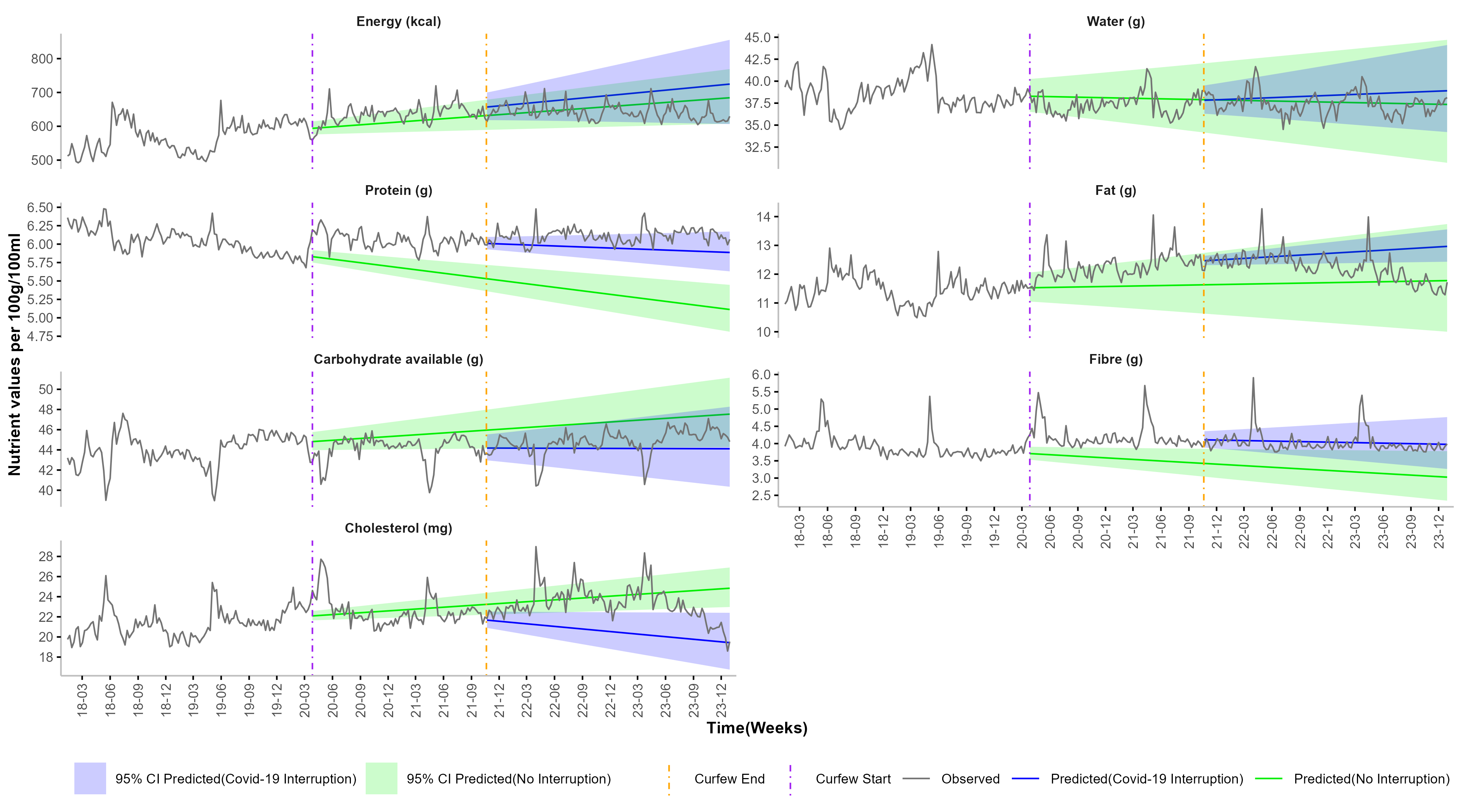

Supplement: S2 Fig — Curfew start vertical line = 27th March 2020 (start of pandemic restrictions). Curfew end vertical line = 20th October 2021 (end of pandemic restrictions). Predictions were estimated in two parts: by extrapolating the pre-pandemic trend and by extrapolating the combined pre-pandemic and pandemic trend. (TIFF) [file pgph.0006544.s011.tiff]

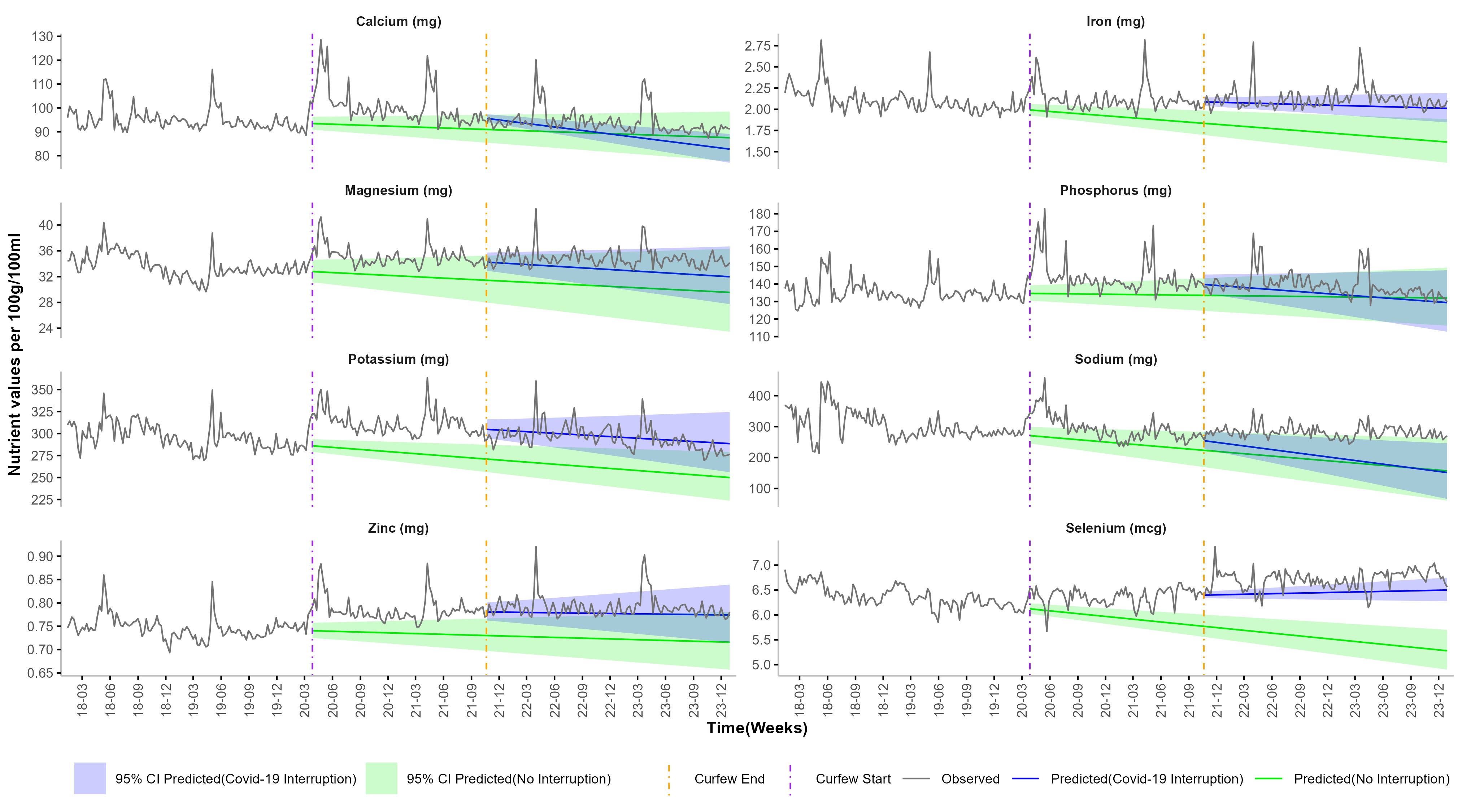

Supplement: S3 Fig — Curfew start vertical line = 27th March 2020 (start of pandemic restrictions). Curfew end vertical line = 20th October 2021 (end of pandemic restrictions). Predictions were estimated in two parts: by extrapolating the pre-pandemic trend and by extrapolating the combined pre-pandemic and pandemic trend. (TIFF) [file pgph.0006544.s012.tiff]

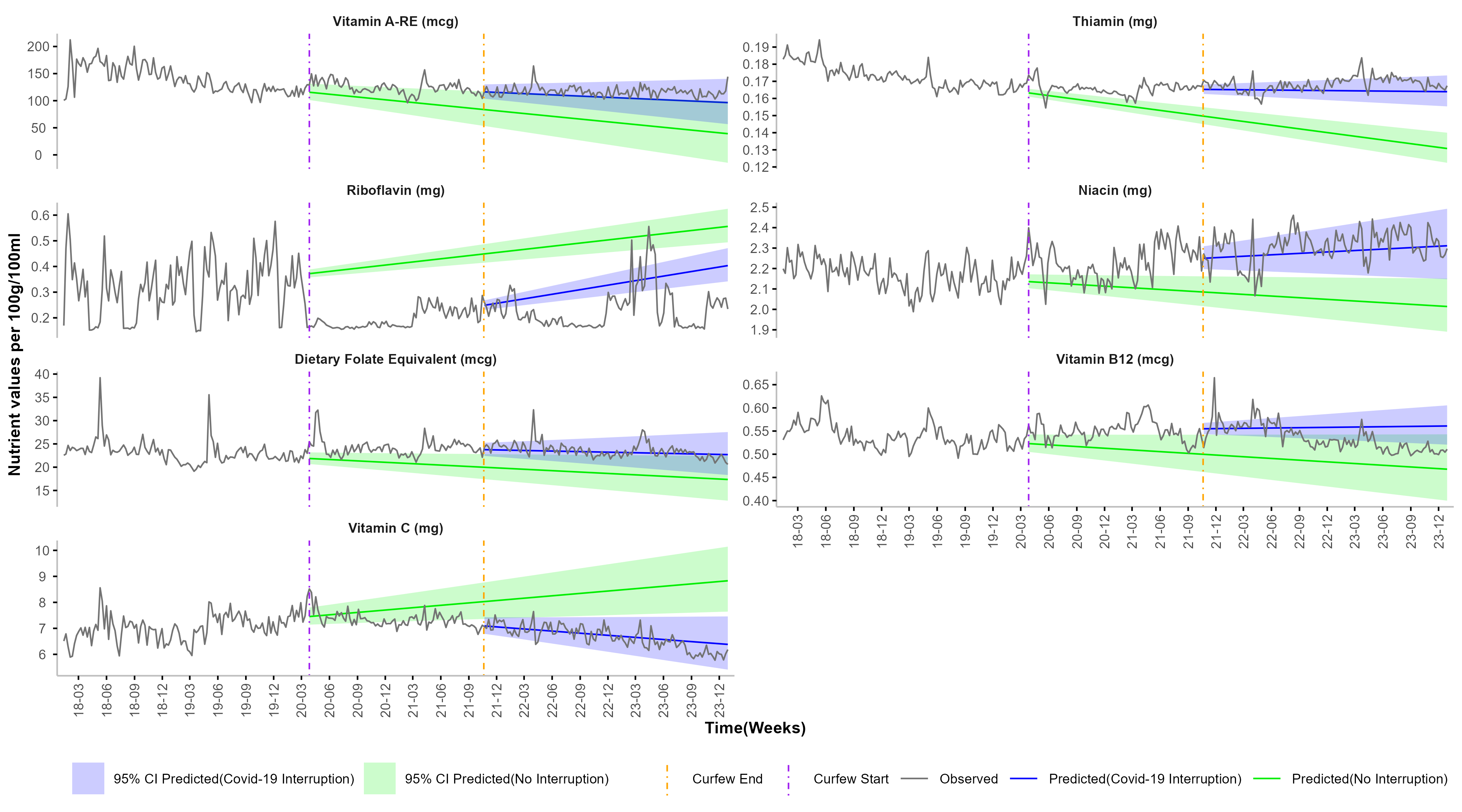

Supplement: S4 Fig — Curfew start vertical line = 27th March 2020 (start of pandemic restrictions). Curfew end vertical line = 20th October 2021 (end of pandemic restrictions). Predictions were estimated in two parts: by extrapolating the pre-pandemic trend and by extrapolating the combined pre-pandemic and pandemic trend. (TIFF) [file pgph.0006544.s013.tiff]
