# Supplementary material for: Food purchase patterns in Nairobi before, during, and after the COVID-19 pandemic lockdown measures
Source: PLOS Glob Public Health. 2026 Jun 1;6(6):e0006544. doi: 10.1371/journal.pgph.0006544 (PMC13225382; doi:10.1371/journal.pgph.0006544)
Supplement: Attachment — Submitted filename: Comments.docx [file pgph.0006544.s014.docx]

**General comments**

The authors should revise the manuscript for typographical and grammatical errors and stick to a particular style of writing. For example, American or British.

**Title**

The title should be written in a consistent style, adhering to the Journal's style, such as sentence case.

**Abstract**

1. The authors should provide the rationale or objective of this study in the background. This information is essential to provide the reader with a clear understanding of the experimental model and the study’s rationale.
2. For clarity and easy readability, authors should include the three periods under study in the methods section.
3. Technically, the reviewer thinks that the results of the study would be more explicit and easier to understand when compared rather than presented for each period individually.
4. The authors should align the conclusion with the objective of the study.
5. Keywords should be arranged alphabetically.

**Introduction**

1. The introduction should be adequately referenced, including lines 50-54.
2. The readership would benefit more if the lockdown situation in Nairobi were also included in the introduction, rather than just the whole of Kenya. It will give perspective to the interpretation of the extent of the challenge and the objective of the study.
3. Authors should reference situations in other African countries to drive home the point.
4. The authors should provide the rationale or objective of this study in the introduction, and also the rationale for selecting only two supermarkets in the city. This information is essential to provide the reader with a clear understanding of the experimental model and the study’s rationale.
5. Finally, authors should include a statement showing the novelty of this study.

**Materials and Methods**

1. Lines 112-113, the authors should include the formal data-sharing agreement as a supplementary file.
2. Authors should avoid discussion in the materials and methods section, for example, lines 142-145, which may not be necessary here.
3. Study design - Authors should state how the ITS was used during the COVID period, as there was no mention of this. Are there any limitations?

**Results and Discussion**

1. Lines 246-268. The figure title captured three periods, but the legends captured two phases. The predictions omit the post-pandemic trend and focus on two parts. Any justification for this?
2. In addition to the separate discussion on the three periods, the authors should have a comparative discussion on the striking peculiarities in the outcomes of these three periods and their implications.
3. Lastly, the authors should avoid the use of the word ‘shift’ consistently in the discussion section and state what the shift is.
4. For easy readability, the authors should separate the recommendations under the conclusion or title it " Conclusion and Recommendations.

**References**

The authors should be consistent in the reference section and address all typographical errors. Stick to a pattern of writing the title of the publication.

**Others**

The figures 1-5 are of poor resolution; the authors should provide clearer images.
