# Supplementary material for: Food purchase patterns in Nairobi before, during, and after the COVID-19 pandemic lockdown measures
Source: PLOS Glob Public Health. 2026 Jun 1;6(6):e0006544. doi: 10.1371/journal.pgph.0006544 (PMC13225382; doi:10.1371/journal.pgph.0006544)
Supplement: Attachment — Submitted filename: Response to Reviewers.docx [file pgph.0006544.s016.docx]

| **Comments** | **How we have Responded to Comments** |
| --- | --- |
| **Journal Requirements/ Editor Comments** | |
| 1. Please amend your detailed Financial Disclosure statement. This is published with the article. It must therefore be completed in full sentences and contain the exact wording you wish to be published.  i. Please clarify all sources of financial support for your study. List the grants, grant numbers, and organizations that funded your study, including funding received from your institution. Please note that suppliers of material support, including research materials, should be recognized in the Acknowledgements section rather than in the Financial Disclosure.  ii. State the initials, alongside each funding source, of each author to receive each grant. For example: "This work was supported by the National Institutes of Health (####### to AM; ###### to CJ) and the National Science Foundation (###### to AM)."  iii. State what role the funders took in the study. If the funders had no role in your study, please state: “The funders had no role in study design, data collection and analysis, decision to publish, or preparation of the manuscript.”  iv. If any authors received a salary from any of your funders, please state which authors and which funders. | Financial Disclosure statement has been amended to “This research was supported by in-house seed funds(Big Ideas Fund) from the African Population and Health Research Center (APHRC) to AK. The funders had no role in study design, data collection and analysis, decision to publish, or preparation of the manuscript.” |
| 2. Please ensure that your Ethics Statement is available in its entirety at the beginning of your Methods section, under a subheading 'Ethics Statement'. | The comment has been implemented |
| 3. For studies involving third-party data, we encourage authors to share any data specific to their analyses that they can legally distribute. PLOS recognizes, however, that authors may be using third-party data they do not have the rights to share. When third-party data cannot be publicly shared, authors must provide all information necessary for interested researchers to apply to gain access to the data. (https://journals.plos.org/plosone/s/data-availability#loc-acceptable-data-access-restrictions)  For any third-party data that the authors cannot legally distribute, they should include the following information in their Data Availability Statement upon submission:  1) A description of the data set and the third-party source  2) If applicable, verification of permission to use the data set  3) Confirmation of whether the authors received any special privileges in accessing the data that other researchers would not have  4) All necessary contact information others would need to apply to gain access to the data | A publicly accessible link to a data repository containing all analysis-specific datasets that can be legally shared has been provided in the manuscript via the Data Availability Statement. “The datasets generated during and/or analysed during the current study are publicly available in the APHRC Microdata Portal Repository via (https://microdataportal.aphrc.org/index.php/catalog/233). The source code used to produce the results and analyses presented in this manuscript is available from GitHub (https://github.com/reinpmomz/Impact-of-COVID_19-on-Food-Purchase-Patterns-Using-ITS).” |
| If the reviewer comments include a recommendation to cite specific previously published works, please review and evaluate these publications to determine whether they are relevant and should be cited. There is no requirement to cite these works unless the editor has indicated otherwise. | This is noted |
| Additional Editor Comments (if provided):  please respond to reviewers' comments | This has been done |
|  |  |
| **Reviewer #1** | |
| **Key recommendations:**  **ESSENTIAL REVISIONS:** |  |
| Systematically replace "intake," "consumption," and "dietary" terminology with "purchased" or "purchasing" throughout the manuscript. This is critical since the study measured purchases, not consumption. | Thank you for highlighting this issue. Where applicable, we have systematically replaced the words "intake," "consumption," and "dietary" with "purchased" across the manuscript as recommended. |
| Address multiple testing: With 162 statistical tests, consider applying corrections (Bonferroni, FDR) or explicitly acknowledge this limitation and emphasize effect sizes. | As defined in the model equation sub-section, our ITS model structure estimates 6 coefficients ($\beta_{0}$, $\beta_{1}$, $\beta_{2}$, $\beta_{3}$, $\beta_{4}$, $\beta_{5}$). These coefficients are estimated within one model. Thus, we run 26 independent ITS models. We have added additional content in the statistical analysis sub-section to place greater emphasis on effect sizes, direction, and p-value significance. |
| Expand discussion of generalizability limitations, particularly regarding socioeconomic bias in supermarket shoppers versus Nairobi's broader population. | We have explicitly acknowledged our limitation in multivariable modeling, as we were not able to explore whether changes in food purchasing differed according to socioeconomic status, region, gender, and age. |
| Better contextualize pre-COVID trends with comparative data from similar urban African settings. | We have added additional content in the discussion section to contextualize pre-COVID and COVID trends from similar urban African settings. |
| **STRONGLY RECOMMENDED:** |  |
| Add discussion distinguishing statistically significant from practically meaningful effects | We have added additional content in the discussion section to explicitly highlight that our interpretation was based on both the statistical significance and the magnitude of the estimated effects. |
| Provide more specific COVID-19 context for Nairobi (case rates, economic indicators, food prices) | We have added additional content in the introduction section to provide more specific COVID-19 context for Nairobi in terms of COVID-19 cases, economic indicators and increase in food prices. |
| Clarify NOVA classification procedures and quality control measures | We have added additional content in the data abstraction and processing sub-section to clarify NOVA classification procedures and quality control measures. |
| Improve figure accessibility (larger fonts, colorblind-friendly colors) | Clear resolution figures have been uploaded as .tiff attachments according to journal requirements. |
| **SUGGESTED:** |  |
| Justify weekly temporal aggregation choice | We have added additional content and citation in the purchase outcomes sub-section to justify weekly aggregation. |
| Expand analysis separating stockpiling from sustained behavioral change | In the Interrupted Time series (ITS) framework, separate parameters were specified to capture the effects of two intervention points: the onset of COVID-19 restrictions and the subsequent lifting of curfews. For the first interruption, the immediate level change was interpreted as capturing short-term behavioral responses, such as temporary stockpiling following the onset of the COVID-19 disruption, while the associated post-interruption slope change represented sustained behavioral adjustments in purchasing patterns during the restriction period. A second set of level and slope parameters was included to model the offset of the intervention following the lifting of curfews, allowing assessment of whether purchasing patterns reverted toward pre-pandemic trajectories or transitioned to a new post-restriction trend. This has been stated in the model equation subsection. |
| Provide model selection details for ARMA orders | We have added additional content in the statistical analysis sub-section to provide model selection details for ARMA orders. |
| Translate key coefficients into interpretable effect sizes | Key coefficients have been translated to interpretable effect sizes, i.e., immediate level change ($\beta_{2}$ and $\beta_{4}$), slope change ($\beta_{3}$ and $\beta_{5}$). A description of the coefficients has been given in the model equation subsection and interpreted in the results section. |
| This is valuable work addressing an important gap in African food systems research with innovative data and rigorous methods.  With the revisions outlined above, it will make a strong contribution to the literature on food purchasing behavior during public health crises. | Thank you for these positive comments. We have revised the manuscript to address the revision comments. |
|  |  |
| **Reviewer #2** | |
| The study has brought some interesting finding, however, it lack to explain the significance of the study on the introduction section which will provide more attention to the readers. | We have added additional content in the introduction section to explain the significance of the study. |
| How was price factors has anlayzed on the food pattern consumption? It would be better to highlight if analysis has been done. | We have added a statement in the model equation sub-section to highlight our study scope, which focused on temporal changes in purchasing behavior and did not explicitly model food price variation. A limitation statement has also been added in the discussion section. |
|  |  |
| **Reviewer #3** | |
| The researchers have used the available data and statistical tools to understand and explain the research. It follow rigorous scientific method for the ETL and data analysis. I didn't find significant concern. | Thank you for these positive comments. |
|  |  |
| **Reviewer #4**  The manuscript on Food purchase patterns in Nairobi before, during, and after the COVID-19 pandemic lockdown measures is relevant and appropriate for the journal. The authors should see the attachment for the necessary comments. | |
| **General comments** |  |
| The authors should revise the manuscript for typographical and grammatical errors and stick to a particular style of writing. For example, American or British. | We have revised the manuscript for typographical and grammatical errors and stuck to American English. |
| **Title** |  |
| The title should be written in a consistent style, adhering to the Journal's style, such as sentence case. | This has been done |
| **Abstract** |  |
| 1. The authors should provide the rationale or objective of this study in the background. This information is essential to provide the reader with a clear understanding of the experimental model and the study’s rationale. | The recommendation has been implemented. |
| 2. For clarity and easy readability, authors should include the three periods under study in the methods section. | We have included the three periods as recommended. |
| 3. Technically, the reviewer thinks that the results of the study would be more explicit and easier to understand when compared rather than presented for each period individually. | The recommendation has been implemented. |
| 4. The authors should align the conclusion with the objective of the study. | Following the addition of recommended content in the background and methods sections, the conclusion aligns with the objective of the study. |
| 5. Keywords should be arranged alphabetically. | This has been done. |
| **Introduction** |  |
| 1. The introduction should be adequately referenced, including lines 50-54. | We have added additional references as recommended. |
| 2. The readership would benefit more if the lockdown situation in Nairobi were also included in the introduction, rather than just the whole of Kenya. It will give perspective to the interpretation of the extent of the challenge and the objective of the study. | We have added additional content to the introduction section to provide a perspective on the lockdown situation in Nairobi. |
| 3. Authors should reference situations in other African countries to drive home the point. | We have added additional content in the introduction section to reference similar situations in other African countries during COVID-19. |
| 4. The authors should provide the rationale or objective of this study in the introduction, and also the rationale for selecting only two supermarkets in the city. This information is essential to provide the reader with a clear understanding of the experimental model and the study’s rationale. | We have added additional content to the introduction to clarify the objective of the study and the rationale for selecting only two supermarkets in the city. |
| 5. Finally, authors should include a statement showing the novelty of this study. | We have added additional content in the Introduction section, showing the novelty of this study |
| **Materials and Methods** |  |
| 1. Lines 112-113, the authors should include the formal data-sharing agreement as a supplementary file. | We did not include the formal data-sharing agreement as a supplementary file because the data are already publicly accessible through the repository link <https://microdataportal.aphrc.org/index.php/catalog/233> provided in the data availability statement. The repository contains the necessary documentation and procedures for requesting and accessing the dataset, making inclusion of the agreement as an additional supplementary file redundant. |
| 2. Authors should avoid discussion in the materials and methods section, for example, lines 142-145, which may not be necessary here. | The content in lines 142–145 was not intended as discussion, but rather to provide a brief methodological context for the interrupted time series (ITS) approach used in this study. This clarification was included to aid readers who may be less familiar with ITS analysis. However, to improve clarity and adhere to conventional structure, we have revised the text to ensure it remains concise and strictly methodological, removing any elements that could be interpreted as interpretative. |
| Study design - Authors should state how the ITS was used during the COVID period, as there was no mention of this.  Are there any limitations? | This has been mentioned in the model equation sub-section.  Limitations have been specified in the discussion section under the strengths and limitations sub-section |
| **Results and Discussion** |  |
| 1. Lines 246-268. The figure title captured three periods, but the legends captured two phases. The predictions omit the post-pandemic trend and focus on two parts. Any justification for this? | The figure legend reflects the model structure, which includes two intervention points: (1) the onset of COVID-19 restrictions and (2) the lifting of curfews. These two interruptions define three distinct time periods, pre-COVID, COVID, and post-COVID, as indicated in the figure title. The legend focuses on the two intervention phases because the Interrupted Time Series (ITS) is parameterized around these interruption points (i.e., level and slope changes at each intervention). In contrast, the three periods in the title describe the resulting temporal segmentation of the series. The predictions do not omit the post-pandemic trend. The post-COVID trajectory is explicitly represented in two ways: (i) the observed post-intervention regression trend following the lifting of curfews (red line), and (ii) the counterfactual trend (dashed blue line, labelled “Counterfactual-COVID-19 interruption”), which extends beyond the curfew end (marked by the dotted dashed orange vertical line). This counterfactual provides the estimated trajectory that would have been expected in the absence of the COVID-19 curfew end. |
| 2. In addition to the separate discussion on the three periods, the authors should have a comparative discussion on the striking peculiarities in the outcomes of these three periods and their implications. | We have revised the comparative framing in the conclusion section to explicitly highlight key differences in the direction, magnitude, and persistence of changes in purchasing behaviour. These revisions improve the overall interpretation of the findings. |
| 3. Lastly, the authors should avoid the use of the word ‘shift’ consistently in the discussion section and state what the shift is. | We have replaced the word “shift” across the discussion section with a more precise language to improve clarity and interpretability for readers. |
| 4. For easy readability, the authors should separate the recommendations under the conclusion or title it " Conclusion and Recommendations. | We have changed the title to Conclusion and Recommendations. |
| **References** |  |
| The authors should be consistent in the reference section and address all typographical errors. Stick to a pattern of writing the title of the publication. | Thank you for highlighting this issue. We used Zotero reference manager, and citation metadata were imported directly from source databases without modification. However, we have thoroughly reviewed the reference section and ensured consistency in formatting across all entries. Publication titles have been standardized to a uniform style, and all typographical errors have been corrected. |
| **Others** |  |
| The figures 1-5 are of poor resolution; the authors should provide clearer images. | Clear resolution figures have been uploaded as .tiff attachments according to journal requirements. |
|  |  |
